# A novel *Penicilliumsumatraense* isolate reveals an arsenal of degrading enzymes exploitable in algal bio-refinery processes

**DOI:** 10.1186/s13068-021-02030-9

**Published:** 2021-09-13

**Authors:** M. Giovannoni, I. Larini, V. Scafati, A. Scortica, M. Compri, D. Pontiggia, G. Zapparoli, N. Vitulo, M. Benedetti, B. Mattei

**Affiliations:** 1 Department of Life, Health and Environmental Sciences, University of L’Aquila, 67100 L’Aquila, Italy; 2 Department of Biotechnology, University of Verona, 37134 Verona, Italy; 3 Department of Biology and Biotechnology “Charles Darwin”, Sapienza University of Rome, 00185 Rome, Italy

**Keywords:** *Penicilliumsumatraense*, Functional genomics, Algal saprophyte, Cell wall-degrading enzymes, *Chlorellavulgaris*, Algal cell wall, Algal bio-refinery, Biofuel

## Abstract

**Background:**

Microalgae are coming to the spotlight due to their potential applications in a wide number of fields ranging from the biofuel to the pharmaceutical sector. However, several factors such as low productivity, expensive harvesting procedures and difficult metabolite extractability limit their full utilization at industrial scale. Similarly to the successful employment of enzymatic arsenals from lignocellulolytic fungi to convert lignocellulose into fermentable sugars for bioethanol production, specific algalytic formulations could be used to improve the extractability of lipids from microalgae to produce biodiesel. Currently, the research areas related to algivorous organisms, algal saprophytes and the enzymes responsible for the hydrolysis of algal cell wall are still little explored.

**Results:**

Here, an algal trap method for capturing actively growing microorganisms was successfully used to isolate a filamentous fungus, that was identified by whole-genome sequencing, assembly and annotation as a novel *Penicillium* *sumatraense* isolate. The fungus, classified as *P.* *sumatraense* AQ67100, was able to assimilate heat-killed *Chlorella* *vulgaris* cells by an enzymatic arsenal composed of proteases such as dipeptidyl- and amino-peptidases, β-1,3-glucanases and glycosidases including α- and β-glucosidases, β-glucuronidase, α-mannosidases and β-galactosidases. The treatment of *C.* *vulgaris* with the filtrate from *P.* *sumatraense* AQ67100 increased the release of chlorophylls and lipids from the algal cells by 42.6 and 48.9%, respectively.

**Conclusions:**

The improved lipid extractability from *C.* *vulgaris* biomass treated with the fungal filtrate highlighted the potential of algal saprophytes in the bioprocessing of microalgae, posing the basis for the sustainable transformation of algal metabolites into biofuel-related compounds.

**Supplementary Information:**

The online version contains supplementary material available at 10.1186/s13068-021-02030-9.

## Background

Microalgae have recently gained increasing interest because of their potential applications in a wide range of industrial sectors, from the biofuel to the pharmaceutical field. Microalgae evolved a great biodiversity, making them a precious source of valuable metabolites such as long chain polyunsaturated fatty acids, vitamins, polysaccharides and proteins [1]. Moreover, microalgae are efficient producers of lipid-rich biomass and have therefore become a key component in sustainable energy development to effectively replace fossil fuels. In this regard, some microalgae species accumulate large amounts of triacylglycerols that, in turn, can be converted to biodiesel through the transesterification process [2]. In addition, microalgae are the bio-factory of first choice for the production of carotenoids with the highest anti-oxidant activity [3]. With the exception of few model species used in basic research, such as *Chlamydomonas* *reinhardtii* [4], the study of microalgal biology is mainly aimed at their exploitation. Knowledge on biological processes such as those related to microalga–saprophyte interactions is still scarce and fragmentary. Similarly to what occurred in the plant–microbe interaction field, in which the study of plant cell walls and microbial cell wall-degrading enzymes (CWDEs) paved the way to the enzymatic saccharification of lignocellulosic wastes for biofuel production [5], so the understanding of degrading processes acted by algivorous and saprophytic microbes towards microalgae can be fundamental for maximizing their use in many applied fields [6].

Indeed, the recalcitrant cell wall of several microalgal species negatively impacts the extractability of triacylglycerol and carotenoids from the algal cell [7, 8], whereas the poor digestibility of microalgae strongly limits their use as additives in feed industry [9, 10]. Moreover, the low extractability of triacylglycerols from microalgae by physical methods negatively impacts the yield of biodiesel from algal-source at industrial scale [11], whereas lipid extraction by chemical (and polluting) methods clashes with the rationale of using microalgae to produce cleaner fuels. Compared to chemical and physical methods employed to break the algal cells, the biological method is an eco-friendlier option and it is easily extendible to large-scale; moreover, the residual biomass from solvent-free extraction techniques can be further valorized by conversion into animal feed or other forms of fuels [12]. To date, the enzymatic treatment of algal cell wall exploited CWDE blends from saprophytic fungi obtained by fermentation processes, in which plant materials are used as feed for the microbes [13–15]. However, these enzymatic arsenals were not evolved to hydrolyse the cell walls of microalgae, resulting in lower degradation efficiencies. In other cases, the enzymatic mixtures consisted of degrading enzymes selected from the most disparate organisms such as chicken, fungi and snails, resulting in highly expensive blends [16, 17] and negatively impacting the production cost of extracted metabolites.

All these aspects prompted us to invest our efforts towards the isolation of novel microbes that can be exploited in the biological treatment of algal biomass. By using an algal trap, we captured a filamentous fungus, later identified as a novel *Penicillium* *sumatraense* isolate, capable of assimilating *Chlorella* *vulgaris*. *Penicillium* is a heterogeneous genus occurring worldwide and its species play important roles as decomposers of organic materials, causing destructive rots in the food industry with the production of a wide range of mycotoxins. Some *Penicillium* species are common indoor air allergens, whereas certain species are considered enzyme factories [18]. Here, an integrated approach of enzymatic assays and liquid chromatography–tandem mass spectrometry (LC–MS/MS) protein analysis revealed the enzymes responsible for the hydrolysis and subsequent assimilation of the oleaginous microalga *C.* *vulgaris* by *P.* *sumatraense*, leading to novel insights that can be useful in algal bio-refinery processes. As a proof of concept, the algalytic activity of the enzymatic mixture from *P.* *sumatraense* was tested for its ability to promote the release of sugars, chlorophylls and lipids from *C.* *vulgaris* and compared to that of other commercial degrading enzymes.

## Results

### Capture of the unknown fungal isolate by the algal trap

**Fig. 1:**
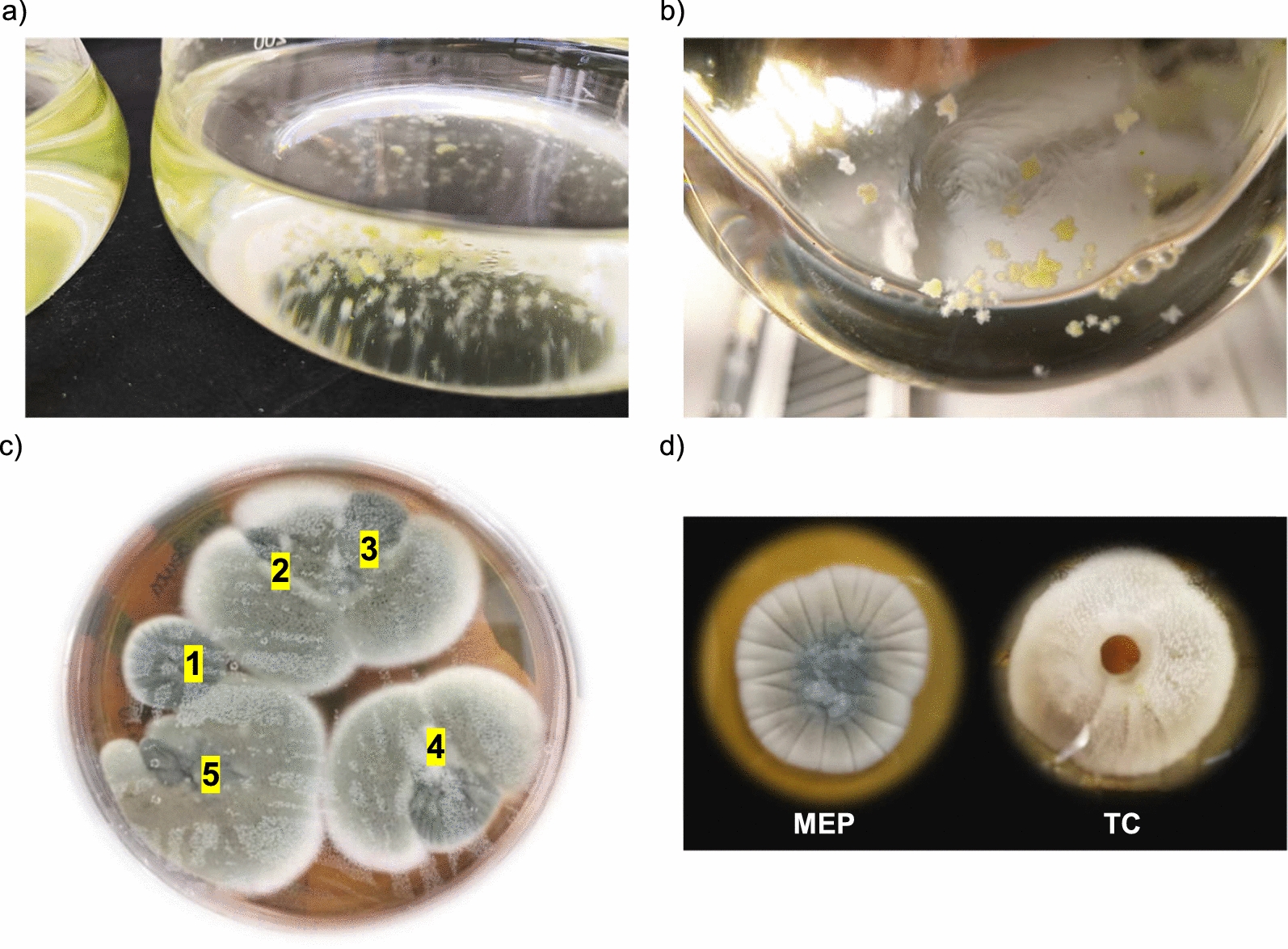
Capture of an unknown fungus by the algal trap. **a**, **b** Capture of the fungus by the algal trap. **b** Details of the mycelium balls from the algal trap shown in **a** upon 6 days of incubation. Trapping experiments were repeated twice with similar results. **c** Isolation of different fungal mycelium portions from the algal trap. Highlighted numbers indicate five different mycelium portions as grown on solid MEP medium. **d** Phenotype of the fungus as grown on solid MEP and TC medium

An algal trap was employed to capture potential algivorous and algal saprophytic microbes. The algal trap consisted of an open flask containing heat-killed cells of *C.* *vulgaris* suspended in T-Phi medium, i.e. TAP medium [19] devoid of acetate in which phosphite (Phi) replaced phosphate (Pi) as phosphorous source (Additional file 1: Figure S1a, b). The algal trap employed *C.* *vulgaris* as organic substrate and Phi as selective agent promoting the isolation of algivorous and algal saprophytic microbes. It is worth noting that Phi is only metabolizable by few chemolithotrophic bacteria possessing the phosphite dehydrogenase enzyme, such as *Pseudomonas* *stutzeri* [20], and therefore an eventual microbial growth in such medium could only be sustained at the expense of *C.* *vulgaris* biomass, used here as carbon and phosphate source. Moreover, Phi is characterized by a mild antimicrobial activity that made the selection in the algal trap more stringent [21–23]. In the absence of antimicrobial agents, several microorganisms could proliferate in the trap eating each other and exploiting the algal-supplemented medium as a mere basal salt source, hindering the discrimination of a real algivorous and algal saprophyte from the rest of contaminating microbes. The algal traps were posed inside a greenhouse, an environment expected to contain plant parasites, saprophytes, endophytic microbes and, at lower extent, phytopathogens. During such attempts, our attention was attracted by a fungus that in two independent trials grew in the traps at the expense of microalgae (Fig. 1a, b). The fungus was able to grow in T-Phi medium exploiting *C.* *vulgaris* as organic substrate, thereby suggesting its capability of metabolizing dead algal biomass. The fungus grew in the form of small mycelium balls capable of adsorbing the microalgae that, in turn, conferred to the mycelium a light green colour (Fig. 1b); for its isolation, different mycelium portions were harvested from the algal traps and plated onto solid MEP medium. All the mycelium portions expanded on the solid medium displaying a similar growth phenotype, suggesting that they could all be ascribed to the same organism (Fig. 1c). The mycelium phenotype was similar to that displayed by fungi belonging to *Penicillium* and *Aspergillus* genus [24, 25] appearing as a greenish or whitish colony based on the type of medium used for growth (Fig. 1d). The morphological characteristics of the colony and conidiophores of the unknown fungal isolate were analysed by microscopy (Additional file 1: Figure S2a, b). The conidiophores were biverticillate as those found in several *Penicillium* species, i.e. whorl of three or more metulae between the end of the stipe and the phialides (Additional file 1: Figure S2b) [18], supporting the identification of the fungus as a *Penicillium* isolate.

### Identification of the unknown fungal isolate by genomic sequencing

The unknown fungal isolate was subjected to whole-genome sequencing for identification. A highly pure genomic DNA preparation was obtained from the mycelium (Additional file 1: Figure S3) and analysed by Next-Generation Sequencing (NGS). A total of more than 290 million reads 2 × 150 bp were obtained. The de novo assembly of the *Penicillium* isolate, obtained by SPAdes [26], was a high-quality draft genome consisting of 129 scaffolds and a size of 35.196.323 bp (scaffolds size ≥ 1000 bp). The GC content was 46.56%, whereas N50 value was 1,455,188 bp in accordance with QUAST statistics [27] (Table 1). It resulted complete and in single copy at 99.3% and 98.2% for fungi and Eurotiales, respectively (Table 2). BUSCO tool [28] quantified the completeness of genomic, transcriptomic or annotated gene datasets in terms of the expected gene content based on evolutionary principles and its metric was complementary to technical metrics like N50 [28]. Regarding gene prediction and annotation, a total of 14,204 genes were predicted by BRAKER2 pipeline [29] trained with protein sequences belonging to *Penicillium* species downloaded from GenBank. PANNZER2 webserver [30] annotations output reported 10,481 proteins with a description and 9854 with at least one Gene Ontology (GO) term.

**Table 1:** QUAST results

	Assembly
#contigs (≥ 0 bp)	4510
contigs (≥ 1000 bp)	129
contigs (≥ 5000 bp)	88
contigs (≥ 10,000 bp)	78
contigs (≥ 25,000 bp)	62
contigs (≥ 50,000 bp)	47
#Total length (≥ 0 bp)	36,393,065
Total length (≥ 1000 bp)	35,196,323
Total length (≥ 5000 bp)	35,103,626
Total length (≥ 10,000 bp)	35,023,964
Total length (≥ 25,000 bp)	34,761,010
Total length (≥ 50,000 bp)	34,215,981
contigs	209
Largest contig	3,498,216
Total length	35,248,380
GC (%)	46.57
N50	1,455,188
N75	808,443
total reads	580,492,180
Mapped (%)	99.21

Genome assembly statistics calculated using QUAST tool. All statistics are based on contigs of size ≥ 500 bp, unless otherwise noted [e.g., “# contigs (≥ 0 bp)” and “#Total length (≥ 0 bp)” include all contigs]

**Table 2:** BUSCO results

	Lineage dataset (_odb10)	No. Sp	Total BUSCO groups searched	Complete BUSCOs (C)	Single-copy BUSCOs (S)	Duplicated BUSCOs (D)	Fragmented BUSCOs (F)	Missing BUSCOs (M)
Genome	Fungi	549	758	753 (99.3%)	753 (99.3%)	0 (0.0%)	0 (0.0%)	5 (0.7%)
Genome	Eurotiales	60	4191	4139 (98.7%)	4117 (98.2%)	22 (0.5%)	13 (0.3%)	39 (1.0%)
Predicted proteins	Fungi	549	758	758 (100%)	749 (98.8%)	9 (1.2%)	0 (0.0%)	0 (0.0%)
Predicted proteins	Eurotiales	60	4191	4164 (99.3%)	4063 (96.9%)	101 (2.4%)	2 (0.06%)	25 (0.7%)

The completeness of genomic, transcriptomic or annotated gene datasets in terms of the expected gene content based on evolutionary principles was quantified by BUSCO (Benchmarking Universal Single-Copy Orthologs) tool. The table displays the completeness of genome assembly and predicted proteins by BRAKER2 based on two datasets (fungi_odb10 and eurotiales_odb10) [No Sp: Number of Species contained in the reference database]

**Fig. 2:**
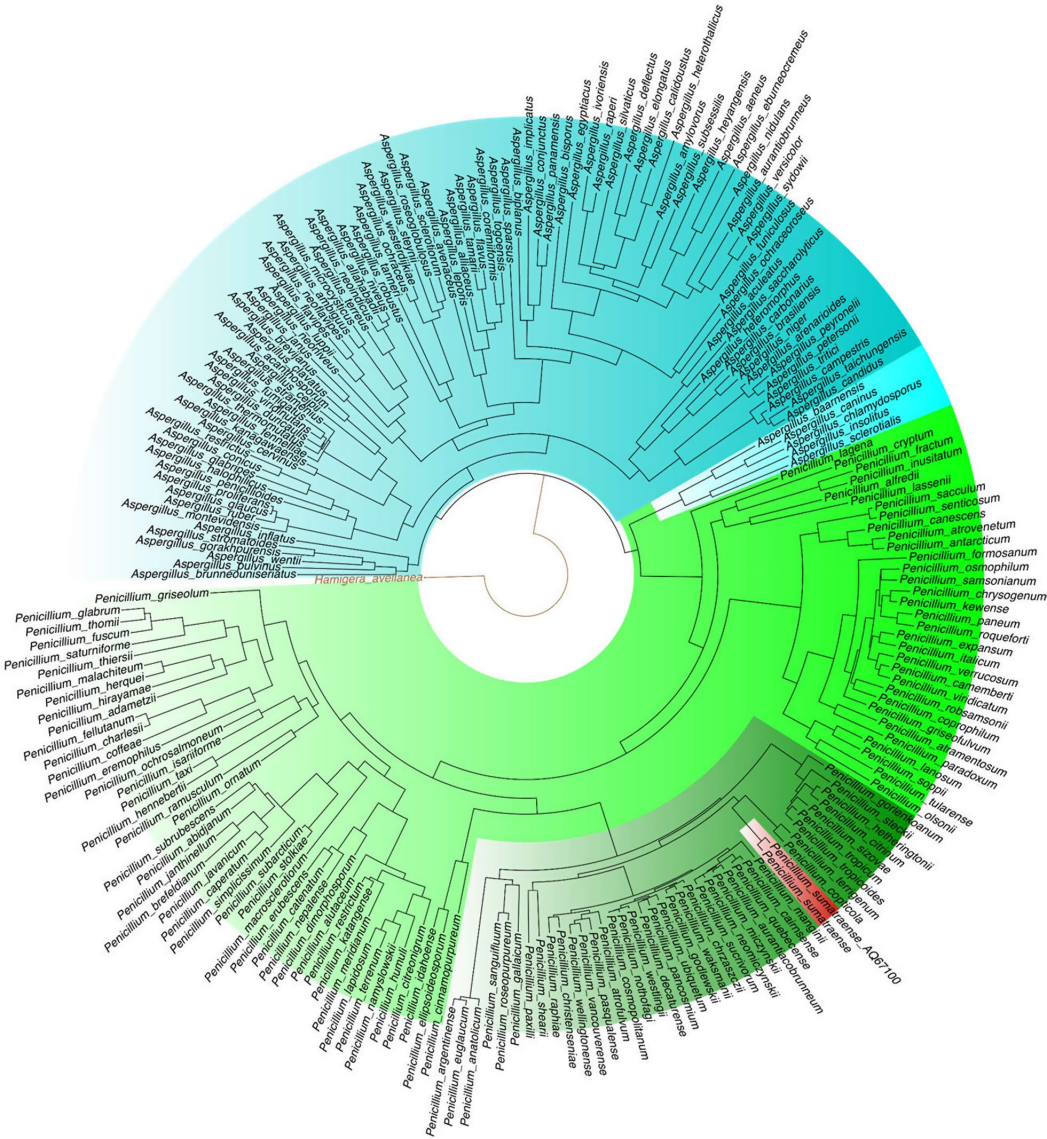
Phylogenetic tree with all *Penicillium* and *Aspergillus* accepted species. The tree shows the phylogenetic proximity of our fungal isolate, i.e. *P.* *sumatraense* AQ67100, with *P.* *sumatraense* (CBS 281.36) (red area). *Aspergillus* and *Penicillium* species are highlighted in cyan and green, respectively, whereas Citrina section is in dark green

**Fig. 3:**
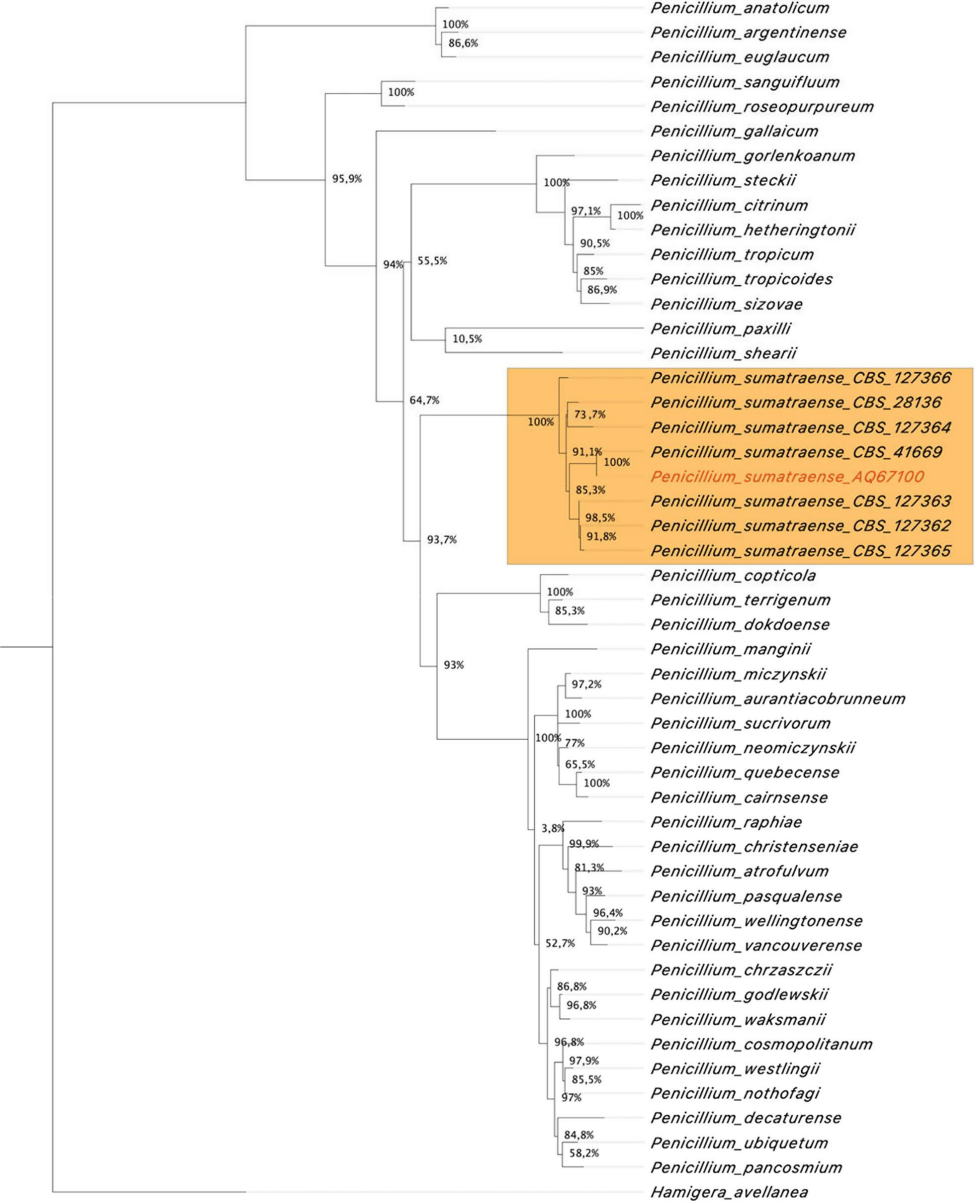
Phylogenetic tree with all *Citrina* *Penicillium* section. The phylogenetic tree shows the clustering of *P.* *sumatraense* AQ67100 with *P.* *sumatraense* CBS strains from *Citrina* section (orange area)

Meanwhile, running BUSCO on predicted proteins, resulted in 100% and 99.3% prediction with fungi and Eurotiales datasets, respectively (Table 2). Other statistics on gene prediction calculated by Eval tool [31] are displayed in Table 3. The sequence of each predicted gene is reported in Additional file 2: Data S1, whereas the corresponding predicted CDS and protein sequences are reported in Additional file 2: Data S2 and S3, respectively. The predicted genes, CDSs and proteins are listed according to incremental numerical identifiers, i.e. gene-, CDS- and protein-IDs, as automatically generated by the BRAKER pipeline; here, each gene and the corresponding CDS and encoded protein are identified by the same number, whereas the suffix “.t” refers to the specific CDS and protein isoform. The structure and coordinates of predicted genes on the genomic sequence are provided in Additional file 2: Data S4. The functional annotations of predicted proteins are shown in Additional file 2: Data S5. A recently published work [25] about the phylogeny of Eurotiales defined the accepted species and strains of *Penicillium* genus, allowing us to construct a phylogeny tree based on the concatenation of four genetic markers, i.e. the genes encoding the RNA polymerase II second largest subunit (*RPB2*), β-tubulin (*BenA*) and calmodulin (*CaM*) together with the Internal Transcribed Spacer (ITS) region (Additional file 2: Data S6). According to the phylogenetic analysis performed by NGPhylogeny [32] with “à la carte” workflow, our isolate was placed near *Penicillium* *sumatraense* (i.e. *Penicillium* *sumatraense* CBS 281.36) and therefore classified as *P.* *sumatraense* AQ67100 on the basis of such result (Fig. 2). A further study on the other *P.* *sumatraense* strains was necessary to understand how they clustered with our isolate. In accordance with previous analysis, *P.* *sumatraense* AQ67100 clustered with the other strains belonging to this species (Fig. 3). *Penicillium* *sumatraense* belongs to series Sumatraensia subgen. Aspergilloides, Citrina section and is phylogenetically related to series Copticolarum [25]. Regarding morphology and physiology, colonies are characterized by moderated or fast growth. Conidial colour ranges from blue green to dull and dark green, and the conidiophores are predominantly biverticillate in accordance with the morphological analysis shown in (Additional file 1: Figure S2). The sexual morphology is unknown and sclerotia are not observed in culture [25]. Moreover, this species produces curvularins such as curvularin, dehydrocurvularin, sumalactone A–D, sumalarins and citridones E–G and it is considered as the causative agent of blue mould rot in *Vitis* *vinifera* and *Sparassis* *crispa* [25].

**Table 3:** Eval statistics

Feature/matric	Genes	Transcripts	Exons	Introns
Count	14,204	14,551	39,734	25,985
Average length	–	1468	473	76.24
Median length	–	1284	255	63
Total length	–	21,364,261	18,797,103	1,981,156
Average coding length	–	1327	–	–
Median coding length	–	1140	–	–
Total coding length	–	19,316,107	–	–
Average exons per transcript	–	2.85	–	–

General statistics on the gene prediction obtained by BRAKER2 using Eval software

### Growth of the fungus in algal-supplemented media

In order to confirm the saprophytic nature of *P.* *sumatraense* AQ67100 towards algal biomass, different mycelium portions were inoculated in two different basal salt media, named as A- and B-medium, whose salt compositions resembled those used for culturing fungi belonging to *Penicillium* and *Aspergillus* genus. Upon 2 days of culturing, heat-treated *C.* *vulgaris* biomass (0.2% w/v) was added to each medium. The use of heat-killed microalgae avoided undesired algal responses that, in turn, could complicate the comprehension of the interaction between the fungus and its feed. Between the two different media, B-medium was the most suitable for supporting the growth of *P.* *sumatraense* AQ67100 in the presence of heat-killed *C.* *vulgaris* cells as substrate (Additional file 1: Figure S4) and therefore it was selected for all the subsequent growth experiments.

**Fig. 4:**
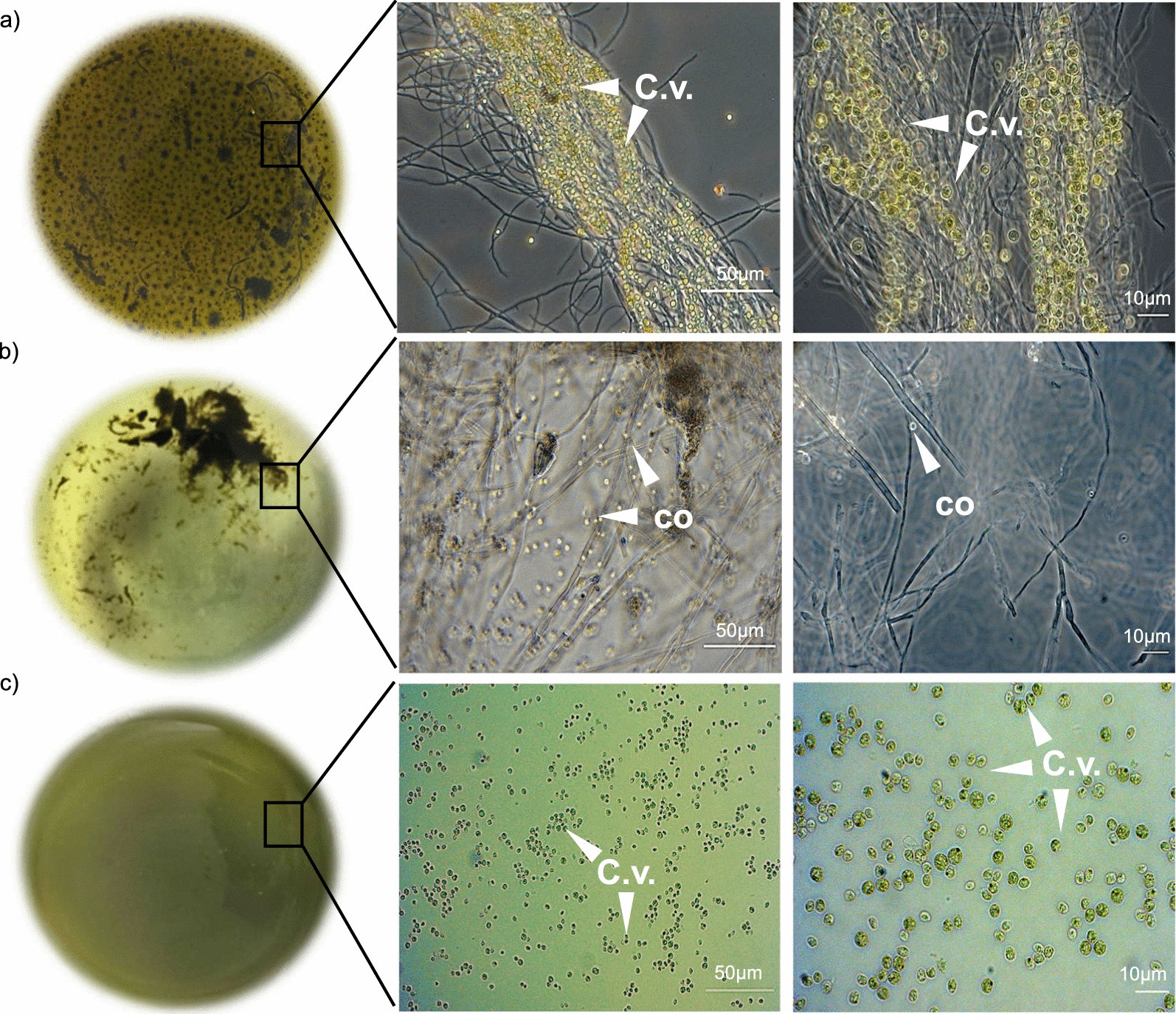
Growth of *P.* *sumatraense* AQ67100 in algal-supplemented medium. **a**, **b** *Left*, representative images of the fungal cultures as grown in B-medium supplemented with 0.2% (w/v) heat-treated *C.* *vulgaris* biomass at **a** 7 and **b** 18 days from algal administration. **c** *Left*, representative image of B-medium supplemented with 0.2% (w/v) heat-treated *C.* *vulgaris* biomass at 18 days from algal administration. **a**–**c** *Middle*-*right*, optical microscope images of the same cultures shown in **a**–**c** *Left.* Scale bars are also indicated [C.v., *C.* *vulgaris* cells; co, conidia]

Four days after algal supplementation, the mycelium started to expand, whereas the green colour of the medium, indicating the presence of microalgae, turned to pale green (Additional file 1: Figure S4b). This effect was clearly visible 7 days after algal supplementation (Additional file 1: Figure S4c); here, the optical microscope analysis revealed that the algal cells were homogeneously adsorbed to the fungal hyphae, indicating a direct contact between the mycelium and dead algal biomass (Fig. 4a). Eighteen days after algal supplementation, the fungus had metabolized most of the microalgae, whereas the amount of mycelium biomass and conidia had increased (Fig. 4b). In the absence of the fungus, the microalgal biomass remained almost unaltered, thus demonstrating that *P.* *sumatraense* was directly responsible for its disappearance from the medium (Fig. 4c). These results indicated that the fungal growth was sustained by the algal biomass since the medium was devoid of any other carbon source, thus demonstrating the saprophytic nature of *P.* *sumatraense* AQ67100 towards algal biomass. The supernatant from the algal-supplemented culture was collected at different days of growth and assayed for the presence of different glycosyl hydrolase (GH) activities. The time-course analysis revealed that the GH activities reached the highest value after 8 days from algal supplementation (i.e. 10-day-old culture) and remained stable for up to 2 days (i.e. 12-day-old cultures). The filtrates from 10-day-old cultures were therefore chosen for all the subsequent analyses. Among the different activities tested, the highest activities were ascribed to β-glucosidase and endo-β-1,3-glucanase and, at lower extent, to endo-β-1,4-glucanase, β-galactosidase and exo-β-1,3-glucanase, while arabinoxylanolytic and xyloglucanolytic activities were almost absent (Additional file 1: Figure S5).

### Analysis of degrading activities of *P. sumatraense* AQ67100 from different polysaccharide- and algal-supplemented media

The degrading arsenal of *P.* *sumatraense* AQ67100 was further investigated by growing the fungus in different polysaccharide- and algal-supplemented media. Different substrates were employed in order to stimulate the production of different categories of CWDEs, since fungi are highly sensitive to the carbon source used for their growth [5]. Culture media were composed of B-medium supplemented with different carbon sources such as heat-treated *C.* *vulgaris* biomass (C.v.), *C.* *vulgaris* cell walls (i.e. alcohol-insoluble solids from *C.* *vulgaris*, abbr. C.v. AIS), arabinoxylan (AX) and xyloglucan (XG). The fungal culture obtained by inoculating the fungus in basal salt B-medium, i.e. without any carbon source, was used as negative control of fungal growth (NS).

**Fig. 5:**
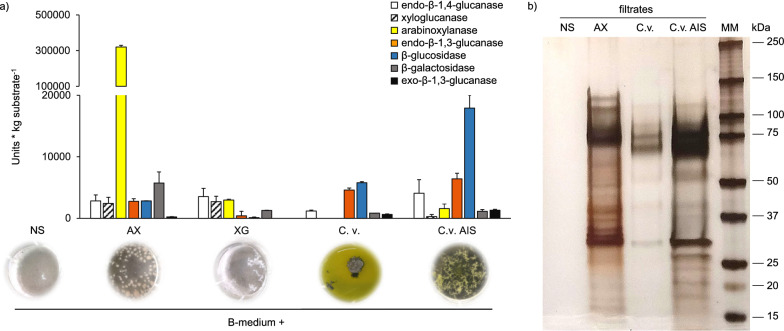
GH activities from *P.* *sumatraense* AQ67100 cultures upon growth in different cell wall polysaccharide-supplemented media. **a** Analysis of GH activities in the filtrates from different cell wall polysaccharide-supplemented cultures upon 10 days of growth. B-medium was supplemented with 0.5% (w/v) arabinoxylan (AX), 0.5% (w/v) xyloglucan (XG), 0.2% (w/v) heat-treated *C.* *vulgaris* biomass (C.v.) and 0.2% (w/v) *C.* *vulgaris* AIS (C.v. AIS). B-medium without any carbon source (NS) was used as negative control. The corresponding cultures are shown below the graph. Units are expressed as µmol reducing ends (for endo-β-1,4-glucanase, xyloglucanase, arabinoxylanase and endo-β-1,3-glucanase activities) and µmol *p*nitrophenol (for β-glucosidase, β-galactosidase and exo-β-1,3-glucanase activities) released per minute. Experiments were repeated in triplicate with consistent results. **B** SDS-PAGE analysis carried out on the same filtrates assayed in **a**. AX and NS filtrates were analysed as positive and negative control of CWDE-production, respectively. The molecular weight marker (MM) is also reported

After 10 days of incubation, the mycelium displayed different growth phenotypes depending on the carbon source used, whereas, as expected, the fungus did not grow on NS medium (Fig. 5a). The supernatants from each culture were collected and the CWDE activities secreted by the fungus were evaluated using different substrates. The enzyme activities were expressed as enzyme units per kg substrate (Fig. 5a) and enzyme units per litre culture (Additional file 1: Figure S6). Notably, *P.* *sumatraense* AQ67100 secreted a high level of xylanolytic activities when the growth was performed using arabinoxylan as carbon source, reaching up to 3.2 × 10^5^ Units kg^−1^ arabinoxylan (Fig. 5a). The use of xyloglucan-supplemented media induced the expression of different GH activities in accordance with the heterogeneous nature of xyloglucan, a β-glucan decorated with d-xylose units and at lower extent, with d-galactose, l-arabinose and d-glucuronic acid residues (Fig. 5a). On the contrary, when the fungus was grown using the cell wall of *C.* *vulgaris* or the whole cells as substrates, cellulolytic and hemicellulolytic activities were significantly lower. Interestingly, β-1,3-glucanase and β-glucosidase activities were the most prominent activity in both algal-supplemented media, suggesting a key role for these GH activities in the cell wall metabolism of *C.* *vulgaris* (Fig. 5a).

Equal volumes of C.v.- and C.v. AIS-filtrates were then analysed by SDS-PAGE including the AX- and NS-filtrates, here used as positive and negative control of enzyme production, respectively (Fig. 5b). The amount of secreted proteins was lower in C.v.-filtrate than in AX- and C.v. AIS-filtrate, in accordance with the level of enzyme activities detected in the same filtrates (Fig. 5a, b).

### Identification of enzymes in the filtrates by LC–MS/MS analysis

**Fig. 6:**
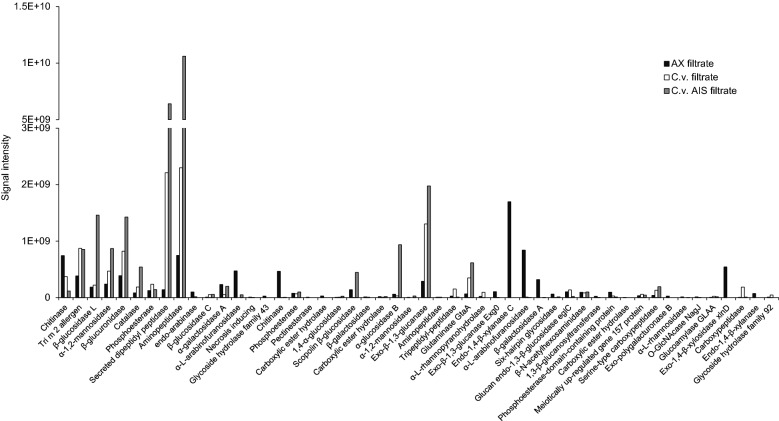
Proteins identified in AX-, C.v.- and C.v. AIS-filtrates as determined by LC–MS/MS analysis. The protein intensity (sum of all identified peptide intensities) and the predicted protein function is reported for each identified protein. Proteins are listed from left to right in decreasing order of sequence coverage. Only proteins identified with the highest score (323.31) are reported. Further details on the identified proteins are reported in Table 4 and Additional file 2: Data S3, S5

Although the enzymatic assays allowed to detect and quantify several enzymatic activities, such analysis was limited by the number of substrates tested. In order to reveal the whole enzymatic arsenal secreted by *P.* *sumatraense* AQ67100, C.v.-, C.v. AIS- and AX-filtrates were also subjected to LC–MS/MS analysis. About 50 proteins were identified at the highest confidence score using a protein database constructed ad hoc from the annotated genome of *P.* *sumatraense* AQ67100 (Table 4). In parallel, protein identification using a database constructed on *C.* *vulgaris* genome [33] did not reveal putative algal proteins in both C.v.- and C.v. AIS-filtrates (data not shown). Interestingly, most of the proteins identified in C.v.- and C.v. AIS-filtrates were related to two main enzymatic classes: proteases and glycosidases (Table 4; Fig. 6). A wide array of proteases was detected in both C.v.- and C.v. AIS-filtrate, suggesting that protein hydrolysis is fundamental to achieve the digestion of *C.* *vulgaris* biomass. Among the different proteases identified, a secreted isoform of dipeptidyl peptidase (g4775.t1, Table 4) and aminopeptidase (g1317.t1, Table 4) were the most prominent in both filtrates (Fig. 6). In order to confirm the presence of proteolytic activities in the filtrates from algal-supplemented cultures, C.v.-filtrate was incubated with BSA; upon 16 h of incubation, BSA resulted almost completely degraded, thus confirming the presence of high proteolytic activity in C.v.-filtrate (Additional file 1: Figure S7).

**Table 4:** Proteins identified in AX-, C.v.- and C.v. AIS-filtrates by LC–MS/MS analysis

Seq. cov. [%]	Unique peptides	MW [kDa]	Protein ID	Predicted protein function
59	13	47.023	g9356.t1	Chitinase
58.9	12	40.258	g10009.t1	Tri m 2 allergen
50.5	20	78.135	g4340.t1	β-glucosidase L
47.4	17	56.436	g1651.t1	α-1,2-mannosidase
43.9	22	71.912	g4150.t1	β-glucuronidase
43.6	24	80.724	g184.t1	Catalase
43.1	11	50.366	g5037.t1	Phosphoesterase
42.1	24	80.26	g4775.t1	Secreted dipeptidyl peptidase
41.4	14	52.692	g1317.t1	Aminopeptidase
39.7	10	36.195	g1453.t1	Endo-arabinase
38.4	15	70.144	g10362.t1	β-Glucosidase C
37.3	16	59.742	g10336.t1	α-Galactosidase A
36.9	15	69.492	g1388.t1	α-l-Arabinofuranosidase
35.4	6	32.237	g6686.t1	Necrosis inducing
**35.1**	**14**	**54.255**	**g6152.t1**	**Glycosidehydrolasefamily43**
34.5	11	43.973	g11244.t1	Chitinase
34.5	11	46.298	g11932.t1	Phosphoesterase
**31.8**	**11**	**42.947**	**g11298.t1**	**Pectinesterase**
**30.1**	**11**	**61.951**	**g9290.t1**	**Carboxylicesterhydrolase**
29.4	8	52.517	g11560.t1	1,4-α-Glucosidase
29.1	22	93.387	g484.t1	Scopolin β-glucosidase
28.9	12	68.34	g1853.t1	β-galactosidase
**28.7**	**10**	**58.956/58.664**	**g8894.t1/t2**	**Carboxylicesterhydrolase**
28.4	20	106.2	g10014.t1	α-glucosidase B
28.3	15	93.568	g9771.t1	α-1,2-mannosidase
28.3	14	85.189	g9376.t1	Exo-β-1,3-glucanase
28.2	24	105.2	g7401.t1	Aminopeptidase
28.2	11	65.349	g12386.t1	Tripeptidyl-peptidase
27.7	12	75.751	g5343.t1	Glutaminase GtaA
27.4	8	51.894	g3293.t1	α-l-Rhamnopyranohydrolase
24.6	15	103.1	g5736.t1	Exo-β-1,3-glucanase Exg0
**24.6**	**7**	**34.711**	**g11633.t1**	**Endo-1,4-β-xylanaseC**
24.3	8	52.419	g411.t1	α-l-Arabinofuranosidase
24.1	16	109.98	g6118.t1	β-galactosidase A
23.9	11	70.86	g8605.t1	Six-hairpin glycosidase
23.9	6	49.312	g7048.t1	Glucan endo-1,3-β-glucosidase eglC
23.8	12	66.558	g3213.t1	β-*N*-Acetylhexosaminidase
23.3	8	49.119	g7605.t1	1,3-β-Glucanosyltransferase
23	10	70.285	g6452.t1	Phosphoesterase-domain-containing protein
22.9	9	61.407	g4908.t1	Carboxylic ester hydrolase
22.8	9	59.375	g10235.t1	Meiotically up-regulated gene 157 protein
22.2	10	64.929	g330.t1	Serine-type carboxypeptidase
**21.9**	**7**	**47.965**	**g10066.t1**	**Exo-polygalacturonaseB**
21.8	14	96.628	g4584.t1	α-l-Rhamnosidase
21.5	10	72.621	g1190.t1	*O*-GlcNAcase NagJ
21.4	6	67.869/68.096	g10052.t1/t2	Glucoamylase GLAA
**21.2**	**14**	**85.962**	**g3950.t1**	**Exo-1,4-β-xylosidasexlnD**
20.4	9	62.198	g8900.t1	Carboxypeptidase
**19.1**	**6**	**42.831**	**g9323.t1**	**Endo-1,4-β-xylanase**
18.2	10	87.891	g9296.t1	Glycosyl hydrolase family 92

The sequence coverage (percentage of the protein sequence covered by identified peptides), unique peptides (number of peptide sequences that are unique to a protein), the protein ID, the expected molecular weight and the predicted protein function are reported for each identified protein. Proteins are listed in decreasing order of sequence coverage. Only proteins identified with the highest score (323.31) are listed. Identification was performed by using the annotated genome of *P.* *sumatraense* AQ67100 as reference database. Proteins specifically identified in AX-filtrate are highlighted in bold. All the remaining proteins were identified in AX- and at least in 1 out of 2 filtrates from algal-supplemented cultures. See Fig. 6 for further details on identified proteins

Other highly represented CWDEs in algal filtrates were glycosidases such as α-glucosidase (g10014.t1, Table 4), β-glucosidases (g4340.t1, g10362.t1, g484.t1, Table 4), α-1,2-mannosidase (g1651.t1, Table 4) and β-glucuronidase (g4150.t1, Table 4). In accordance with the GH activities previously detected (Fig. 5a, Additional file 1: Figure S6), four enzymes related to the metabolism of β-1,3-glucan were also identified (g9376.t1, g5736.t1, g7048.t1, g7605.t1, Table 4) with an exo-β-1,3-glucanase (g9376.t1, Table 4; Fig. 6) being the most prominent in C.v. AIS-filtrate (Fig. 6). Other endo-acting enzymes that are usually required for the efficient hydrolysis of plant cell walls, such as endo-β-1,4-glucanases, endo-β-1,4-xylanases and endo-α-1,4-polygalacturonases, were hardly detected (Table 4; Fig. 6).

The molecular weights of the most prominent enzymes from C.v.- and C.v. AIS-filtrates (Table 4; Fig. 6) such as the exo-β-1,3-glucanase (g9376.t1, 85.189 kDa), secreted dipeptidyl peptidase (g4775.t1, 80.26 kDa), β-glucosidase L (g4340.t1, 78.135 kDa), β-glucuronidase (g4150.t1, 71.912 kDa) and aminopeptidase (g1317.t1, 52.692 kDa) were compatible with those of the protein bands revealed by the SDS-PAGE analysis shown in Fig. 5b (Additional file 1: Figure S8). However, except for a ~ 55-kDa band that could be ascribed to the aminopeptidase, other bands corresponding to individual enzymes in the 60–90 kDa range could not be clearly resolved due to the heterogeneous composition of the raw filtrates of *P.* *sumatraense* AQ67100 that included many different proteins with similar molecular weights (Table 4; Additional file 1: Figure S8).

In AX-filtrate, in addition to glycosidases and proteases (Fig. 6), several xylanolytic enzymes such as endo-xylanases (g11633.t1, g9323.t1, Table 4; Fig. 5), β-1,4-xylosidases (g3950.t1, Table 4; Fig. 6) and α-l-arabinofuranosidases (g1388.t1, g411.t1, Table 4; Fig. 6) were specifically identified (Table 4).

The ubiquitous presence of two chitinases in AX-, C.v.- and C.v. AIS-filtrates (g9356.t1, g11244.t1, Table 4; Fig. 6) suggested their involvement in endogenous processes such as the remodelling of fungal hypha rather than in the degradation of specific algal chitin-like polysaccharides [14, 15]. The role of chitinases secreted by *P.* *sumatraense* AQ67100 was further investigated by growing the fungus in a chitin-supplemented medium (Additional file 1: Figure S9). After 10 days of incubation, a stunted mycelial growth together with a high amount of residual substrate indicated that crystalline chitin is not an ideal substrate for *P.* *sumatraense* AQ67100 (Additional file 1: Figure S9a) since the chitinolytic activity of *P.* *sumatraense* AQ67100 did not efficiently hydrolyse chitin of exogenous origin. Moreover, chitin did not induce the production of any relevant GH activity except for a slight increase of endo-β-1,3-glucanase activity (Additional file 1: Figure S9b).

The lack of degrading enzymes exclusively induced by algal supplementation together with the high propensity in the production of xylanolytic activities suggested that arabinoxylan is a preferred substrate for *P.* *sumatraense* AQ67100 compared to algal biomass. However, *P.* *sumatraense* AQ67100 can be considered a versatile saprophyte in accordance with the biological role of this category of microbes.

### Enzymatic treatments of *C. vulgaris* using the filtrate of *P. sumatraense* AQ67100

In order to evaluate the degrading potential of *P.* *sumatraense* towards algal biomass, the filtrate of *P.* *sumatraense* AQ67100 from the algal-supplemented culture, referred to as F-blend, was used to treat *C.* *vulgaris* cells*.* Other degrading enzymes such as a pure cellulase, referred to as C-blend, and an enzyme mixture composed of lysozyme, chitinase and sulfatase, referred to as LCS-blend, were used in control experiments. Indeed, peptidoglycan-degrading enzymes such as lysozyme have been recently shown to be highly effective towards the cell wall of *C.* *vulgaris* [34], and the LCS-blend was also employed to obtain *C.* *vulgaris* protoplasts [17]. The amount of sugars, chlorophylls and lipids released from the enzymatically treated cells was determined in the incubation medium upon 16 h of treatment. Differently from sugars, that may also have a cell wall origin, an increased release of chlorophylls and lipids in the supernatants can be considered as a direct proof of cell lysis, given the intracellular compartmentalization of these metabolites. In parallel, the amount of chlorophylls and lipids was also evaluated in the ethanolic extracts from the same cells. It is worth noting that in normal conditions (i.e. untreated cells), metabolite extraction by 60% ethanol is not efficient for *C.* *vulgaris*.

**Fig. 7:**
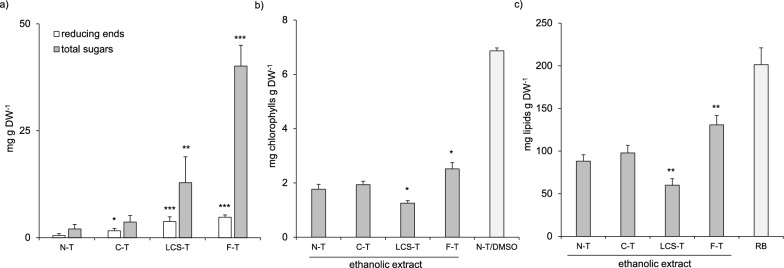
Algal treatment using *P.* *sumatraense* AQ67100 filtrate improves the release of metabolites from *C.* *vulgaris*. **a** Sugars released in the incubation medium from enzymatically treated *C.* *vulgaris* cells. **b**, **c** Amount of **b** chlorophylls and **c** lipids in ethanolic extracts from enzymatically treated *C.* *vulgaris* cells. [N-T: no treatment; C-T: treatment with cellulase blend; LCS-T: treatment with lysozyme–chitinase–sulfatase blend; F-T: treatment with P*.* *sumatraense* blend; N-T/DMSO: no treatment + DMSO-extraction; RB: total lipids from raw algal biomass]. Data are expressed as mean ± SD (N ≥ 5). Asterisks indicate statistically significant difference against control (N-T) according to Student’s t test (*p ≤ 0.005; **p ≤ 0.0005; ***p ≤ 0.0001)

Although at different extent, all the enzyme blends promoted the release of sugars (Fig. 7a), whereas none of the treatments was able to promote the release of chlorophylls and lipids in the incubation medium (data not shown), indicating that the released sugars are likely degradation products of cell wall components rather than compounds of intracellular origin. Among the different blends, the highest release of reducing and total sugars (4.8 ± 0.5 mg g DW^−1^, 40.1 ± 4.9 mg g DW^−1^) was promoted by F-blend, followed by LCS-blend (3.7 ± 1.1 mg g DW^−1^, 12.9 ± 6 mg g DW^−1^), whereas C-blend had very little effect (Fig. 7a). Compared to the control experiment (N-T), F-blend was the only mixture capable of promoting an increased release of both chlorophylls (2.5 ± 0.2 mg g DW^−1^) and lipids (130.7 ± 10.9 mg g DW^−1^) from algal biomass upon ethanolic extraction, thus demonstrating the effectiveness of *P.* *sumatraense* AQ67100 filtrate in increasing the permeability of *C.* *vulgaris* cells (Fig. 7b, c). On the other hand, the treatment with LCS-blend lowered the extraction yield of both chlorophylls and lipids with respect to that obtained from untreated cells (Fig. 7b, c), suggesting the presence of unexpected side-reactions between LCS-blend and the cells of *C.* *vulgaris*. The latter result highlighted the importance of selecting the proper enzyme blend for algal bio-refinery processes that should be effective towards cell wall components and compatible with downstream processing of algal biomass.

## Discussion

In the present research, a filamentous fungus capable of metabolizing *C.* *vulgaris* was captured by an algal trap (Fig. 1) and classified as *P.* *sumatraense* AQ67100 by genomic analysis (Figs. 2, 3). Although different *Penicillium* species have been widely investigated, the current available information on *P.* *sumatraense* is still scarce. The first isolate was obtained from the rhizosphere of the mangrove *Lumnitzera* *racemose* [35]; subsequently, another isolate from deep-sea sediments was shown to produce sumalactones, a new class of curvularin-type macrolides [36]. In 2017, *P.* *sumatraense* was identified as one of the fungi responsible for blue mould disease in *V.* *vinifera* [37]. Recently, the use of *P.* *sumatraense* as a bioreactor for lipase production has been proposed [38]. To our knowledge, the genome of *P.* *sumatraense* AQ67100 is the first fully annotated genome available for this fungal species.

Although *P.* *sumatraense* AQ67100 showed a high propensity in the production of xylanolytic activities in accordance with other fungal species belonging to the same genus [39, 40], our isolate metabolized *C.* *vulgaris* by a combined action of different glycosidases and proteases (Figs. 5, 6; Table 4). The array of enzyme activities secreted by *P.* *sumatraense* AQ67100 towards the cell walls and whole cells of *C.* *vulgaris* did not resemble the conventional degradative activities required for the degradation of plant cell wall polysaccharides. Amongst the different CWDEs secreted in the algal-supplemented media, glycosidases were the most represented GHs (Fig. 6; Table 4). Glycosidases are highly versatile enzymes, employed in several industrial sectors due to their broad substrate specificity. Presumably, *P.* *sumatraense* AQ67100 could exploit the versatility of these enzymes towards the heterogeneous cell wall of *C.* *vulgaris*. Proteases were identified both in C.v.- and C.v. AIS-filtrates, indicating that their degrading activity is directed not only towards proteins released from the disrupted cells, but also towards structural proteins of the algal cell wall. Notably, glycoproteins are structural cell wall components in several microalgal species [41, 42] and different works demonstrated that the treatment of *C.* *vulgaris* biomass with commercial proteases improved its assimilation by methanogenic bacteria, resulting in higher biogas yield [43–45]. In addition to proteases and glycosidases, our results showed that β-1,3-glucan hydrolases may also play an important role in the degradation of *C.* *vulgaris* biomass (Figs. 5, 6; Table 4). It is worth noting that β-1,3-glucan is a major cell wall component of brown seaweed [46] and microalgae including *C.* *vulgaris* [34, 47, 48], and in higher plants it is also known as callose, a defence-induced polysaccharide highly recalcitrant to CWDE hydrolysis [49]. Interestingly, a β-1,3-glucanase-encoding gene is conserved across 27 different Chlorella viruses, thereby suggesting the involvement of this enzyme in the degradation of the host cell wall either during virus release and/or entry [50]. However, the characteristics of polysaccharide accessibility in the *C.* *vulgaris* cell wall cannot be inferred based only on the enzymes used by *P.* *sumatraense* AQ67100*.* Other algal saprophytes may employ different enzymes since the arsenal of CWDEs also reflects host preference [51], pointing to the necessity of integrating different microbial secretomes for a more comprehensive scenario. Moreover, ultra-structural, NMR and monosaccharide composition analyses will be fundamental to accurately decipher the complex structure of *C.* *vulgaris* cell wall.

During the first days of incubation with the algal biomass, the fungal hyphae attracted the dead algal cells to their surface (Fig. 4a). For certain aspects, such interaction could resemble a fungal-assisted flocculation event, a phenomenon observed in different fungi–microalgae interactions and proposed as a potential eco-friendly harvesting process [52–54]. However, at longer incubation times, *P.* *sumatraense* AQ67100 clearly grew at the expense of *C.* *vulgaris* (Fig. 4b; Additional file 1: Figure S4). The adhesion of fungal hyphae to *Chlorella* cells could also evoke a epibiotic or endobiotic style of predation [55], similar to that displayed by *Vampirovibrio* *chlorellavorus* [56]. One aspect that must be taken into account is the tendency of fungi to adhere to organic substrates as well as to inert surfaces [57]. In our experiments, the use of dead cells instead of living microalgae focused the analysis on the saprophytic nature of the fungus rather than on its potential predator and pathogenic nature; therefore, further studies will be required to investigate the interaction between *P.* *sumatraense* AQ67100 and living cells of *C.* *vulgaris*.

Although the treatment with two out of three enzymatic blends promoted the release of sugars from the cells of *C.* *vulgaris* (Fig. 7a), the filtrate from *P.* *sumatraense* was the only blend capable of promoting a higher release of chlorophylls and lipids upon ethanolic extraction (Fig. 7b, c). The lipid-rich biomass of *Chlorella* species has attracted considerable interest, due to its potential application as a renewable and sustainable source of biofuel. However, biological constraints still pose significant challenges to the development of economically viable large-scale production of algal biofuel.

**Fig. 8:**
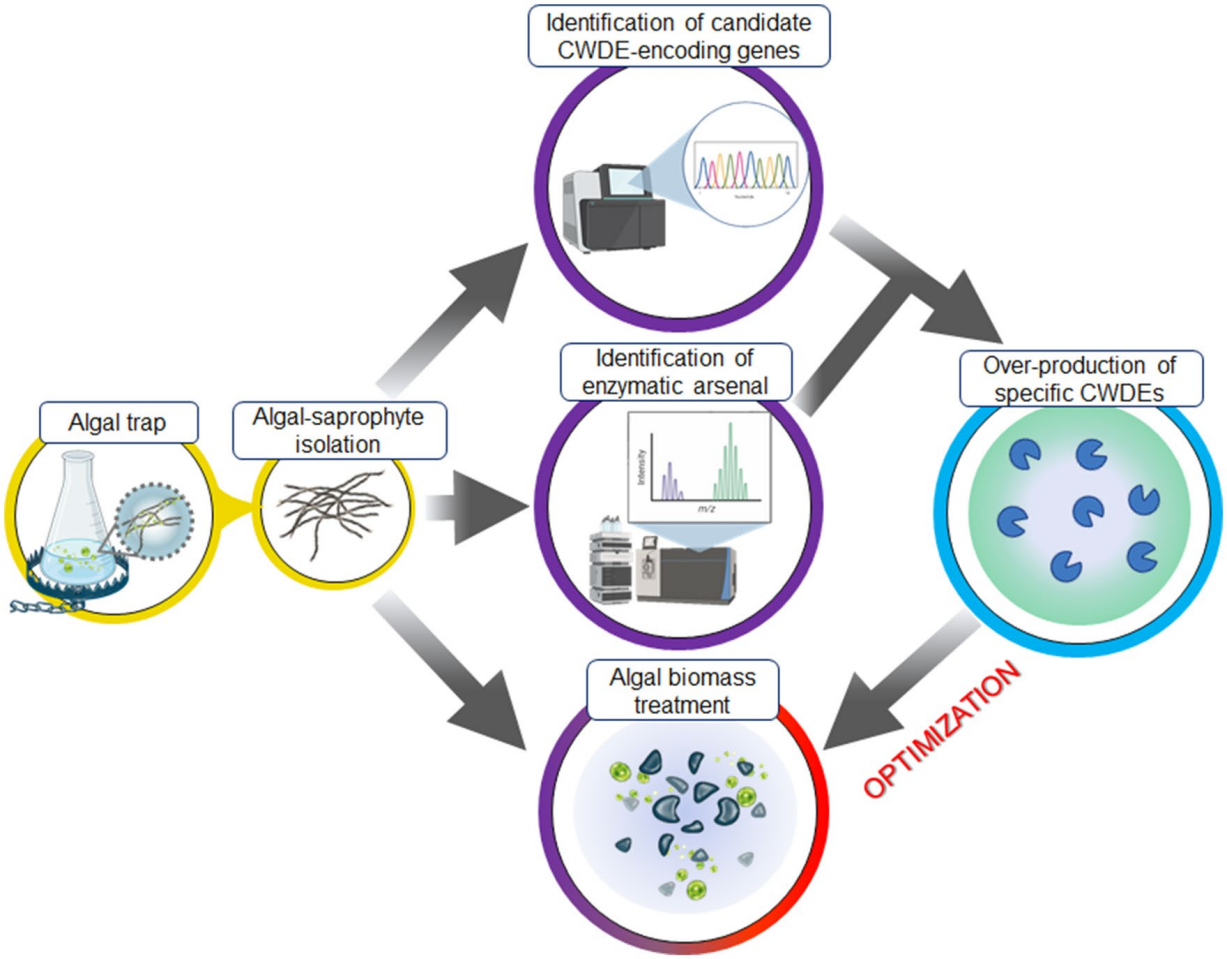
Biological treatment of algal biomass by enzymatic blends from algal saprophytes. Schematic representation of the workflow for the design of algalytic formulations using the algal trap approach

In this regard, the low efficiency of metabolite extraction is one of the main drawbacks that still limit a sustainable use of microalgae in the biofuel sector. Compared to the untreated algal biomass, the treatment with the fungal filtrate improved the release of chlorophylls and lipids by 42.6 and 48.9%, respectively. Based on our results (Fig. 7), at least ~ 4.7 L of fungal culture are required to efficiently extract all the lipids from one gram of *C.* *vulgaris* biomass. Except for xylanolytic activities (3.2 × 10^5^ Units kg arabinoxylan^−1^), the level of other CWDE activities secreted by *P.* *sumatraense* AQ67100 was quite low (Fig. 5a), highlighting the importance of finding optimal growth conditions to fully exploit the potential of this fungus in industrial processes. Importantly, the activity displayed by the fungal filtrate towards *C.* *vulgaris* seemed more permeabilizing than algalytic, indicating that the cell wall of *C.* *vulgaris* was degraded only in part. In this regard, the enzymatic hydrolysis of *C.* *vulgaris* will require further optimization, e.g., by testing different pH values, temperature conditions, reaction times, substrate concentrations and enzyme/substrate loadings. However, our analyses revealed several degrading enzymes exploitable in algal processing that can be heterologously expressed at high level for further characterization and large-scale exploitation, e.g., as supplements in specific algalytic blends (Table 4; Fig. 8) [17, 34].

Based on the characterization of the enzymatic profiles in *P.* *sumatraense* AQ67100 secretome, this fungus cannot be considered as a specialized algal saprophyte, but rather as an opportunistic saprophyte capable of assimilating microalgae in case of necessity, i.e. when the microalgae are the unique available carbon source in the medium (Fig. 5). The enzymes used to assimilate *C.* *vulgaris* were not exclusively induced by algal biomass (Table 4), in contrast to the activities specifically secreted by *P.* *sumatraense* AQ67100 for arabinoxylan degradation. However, our results indicate how the fungus remodulated its enzymatic arsenal in relation to *C.* *vulgaris*, pointing to proteases, β-1,3-glucanases and glycosidases as the main enzymes responsible for algal assimilation. Furthermore, the catabolic reactions of chitin-like polysaccharides acted by *P.* *sumatraense* AQ67100 will require a deeper investigation (Fig. 6; Table 4; Additional file 1: Figure S9b). The latter aspect is particularly important if we consider that at present, the industrial use of chitinases is still limited by the lack of efficient chitinase-producing biofactories [5].

It seems likely that the isolation of a facultative algal saprophyte using the algal trap could depend on the location of the trap, in this case a greenhouse mostly used for growing land-plants such as *Nicotiana* *tabacum*, *Lycopersicon* *esculentum* and *V.* *vinifera*. A further improvement of this approach will consist in positioning the algal trap in proximity of algal open-ponds and marshy areas in order to increase the probability of capturing algivorous organisms and specialized algal saprophytes.

## Conclusions

The algal trap approach allowed to identify the enzymatic arsenal exploited by a novel *P.* *sumatraense* isolate to assimilate *C.* *vulgaris*. The enzymatic arsenal was composed of proteases such as dipeptidyl- and amino-peptidases, β-1,3-glucanases and glycosidases including α- and β-glucosidases, β-glucuronidase, α-mannosidases and β-galactosidases. Notably, the enzyme mixture increased the efficiency of extraction of both chlorophylls and lipids from *C.* *vulgaris,* highlighting the potential of microalga-microbe research in the bioprocessing of useful metabolites from microalgae (Fig. 8). The use of algal saprophytes in biomass pretreatment should be comprehensively evaluated taking into consideration the lipid extraction efficiency, scalability and overall cost of the extraction process, with the aim to improve the competitiveness of algal-derived biofuels.

## Methods

### Capture of *P. sumatraense* by the algal trap

The algal trap consisted of an open flask containing 5 × 10^8^ heat-killed *C.* *vulgaris* cells suspended in 200 mL of T-Phi medium. The flask opening was covered by a square plastic mesh (4 mm^2^ hole) in order to avoid the entrance of macroscopic contaminants. T-Phi medium was a modified version of TAP medium, i.e. TAP medium devoid of acetate and supplemented with 0.7 mM KH_2_PO_3_ (Phi) instead of 1.2 mM PO_4_^3−^ (Pi) [19]. Phi was purchased from Wanjie Int., China (CAS No. 13977-65-6). The medium was prepared fresh, pH-adjusted (6.9) and autoclaved. The heat-treatment of *C.* *vulgaris* was performed by incubating the cells at 70 °C for 50 min.

For each trial, four traps were posed inside a greenhouse on a rotary shaker (150 rpm). After 5 days, the traps were visual analysed to detect eventual contaminants. Macroscopical fungal contaminants were isolated by transferring different mycelium portions onto solid MEP medium [2% (w/v) Malt-agar, 1% (w/v) Peptone, 1.5% (w/v) micro-agar, 100 µg mL^−1^ ampicillin] and incubated at 20 °C in a dark chamber. The fungal isolate, later identified as *P.* *sumatraense,* was maintained at 20 °C in a dark chamber on solid MEP or TC medium [1% (w/v) d-glucose, 1.7% (w/v) malt-agar, 0.1% (w/v) asparagine, 0.001% (w/v) thiamin, 0.2% (w/v) KH_2_PO_4_, 0.2% (w/v) yeast extract, 0.1% (w/v) MgSO_4_, 1.5% (w/v) micro-agar].

### Algal strain and culture conditions

*Chlorellavulgaris* wild-type strain 211-11p was obtained from the Culture Collection of Algae (Göttingen University, Germany). *C.* *vulgaris* was maintained at 26 °C, with a 16/8 h light/dark photoperiod, light intensity of 35 µmol photons m^−2^ s^−1^, in solid or liquid TAP medium on a rotary shaker (180 rpm) according to [22]. Growth was followed by measuring cell density using an automated cell counter (Countess II FL Cell Counter, ThermoFisher). *C.* *vulgaris* cells from the stationary growth phase were used in all the experiments.

### Morphological analysis

The morphological characteristics of the mycelium were investigated by microscopy analysis using a stereo microscope (Leica S8-Apo) equipped with EC3 camera (8X magnification) or a phase contrast ZeissAxio Imager A2 (100× magnification, oil immersion) equipped with a colour microscope camera (Leica DFC 320 R2). For optical microscope analysis, the mycelium was harvested from the algal-supplemented medium and fixed using an 85% (w/v) d-lactic acid solution (Sigma-Aldritch) as mounting fluid.

### Genomic extraction and NGS analysis

For genomic extraction, 1–2 g of fresh mycelium were harvested from solid MEP medium and grinded in liquid nitrogen. The grinded sample was transferred into a 50 mL tube containing 6 mL of extraction buffer (7 M Urea, 0.3 M NaCl, 0.02 M EDTA, 0.03 M *N*-lauroyl-sarcosine, 0.05 M Tris–HCl pH 8.0). Then, 3 mL of phenol and 3 mL of chloroform: isoamyl-alcohol (24:1) were added to the sample and vortexed. Upon centrifugation (10,000×*g*, 10 min), the upper phase was transferred into a fresh tube by adding 3 mL of 7.5 M NH_4_CH_3_CO_2_ and 3.6 mL of isopropanol. Upon mixing and centrifugation (10,000×*g*, 5 min), the pellet was washed with 70% (v/v) ethanol, air-dried and then dissolved in 0.25 mL of ultrapure water. The genomic preparation was subjected to NGS analysis by Illumina NovaSeq technology at Dante Labs (L’Aquila, Italia; https://dantelabs.it/pages/our-labs) and the sequencing results analysed by bioinformatic tools.

### Genome assembly, annotation and phylogenetic classification

Illumina paired end sequences were trimmed with Trimmomatic v0.36 [58] and the reads were assembled with SPAdes genome assembler v3.11.1 [26] using default parameters. The assembly obtained was quality assessed with QUAST v5.0.2 [27] and BUSCO (Benchmarking Universal Single-Copy Orthologs) v4.1.4 [28] in order to evaluate the completeness. QUAST was run with k-mer-based quality metrics and ribosomal RNA genes prediction options and BUSCO with fungi_odb10 and eurotiales_odb10 lineage options. The whole ribosomal region was reconstructed by using the fragmented ribosomal sequences retrieved in the assembly by ITSx tool v.1.1.1 [59] as seed for the tool GetOrganelle v1.6.4 [60]. The reconstructed ribosomal sequences, containing the full ITS, were extracted by the software ITSx v1.1.2 [59]. The ITS sequence, composed of ITS1, 5.8S and ITS2, was used as a query for a BLASTn [61] search against NCBI (National Center for Biotechnology Information) non redundant database. All the matches with query coverage and identity from 99 to 100% belonged to *Penicillium* genus. For this reason, all *Penicillium* proteins (444,543 sequences in October 14, 2020) were downloaded from NCBI and used as external evidence to annotate the genome with BRAKER2 v2.1.5 pipeline [29]. BRAKER2 is an extension of BRAKER1 [62], which allows fully automated training of the gene prediction tools GeneMark-EP + [63] and AUGUSTUS [64] from RNA-Seq and/or protein homology information, and it integrates the extrinsic evidence into the prediction. Moreover, BRAKER2 [29] reaches high gene prediction accuracy even in the absence of the annotation of very closely related species and in the absence of RNA-Seq data (EP-mode). So, the pipeline was run in EP-mode, fungus option activated and *Penicillium* *sumatraense* as species selected, i.e. the ITS best BLASTn hit. The genome in input for BRAKER2 was repeat masked with a de novo repeat finding program, i.e. RepeatModeler v1.0.11 (http://www.repeatmasker.org/RepeatModeler/). Predicted proteins were functionally annotated using PANNZER2 (Protein ANNotation with Z-scoRE), a rapid functional annotation server [30].

As described by Houbraken and colleagues in a recent study [25], four phylogenetic markers were used to classify *Aspergillus* and *Penicillium* species, i.e. the genes encoding β-tubulin (*BenA*), calmodulin (*CaM*) and RNA polymerase II second largest subunit (*RPB2*) together with the Internal Transcribed Spacer (ITS) region. Therefore, in order to identify with more accuracy our isolate *Penicillium*, a phylogenetic analysis was performed concatenating the nucleotide sequences of these genes into one unique sequence. Then, all the markers from *Aspergillus* and *Penicillium* accepted species already described [25] were downloaded from NCBI and concatenated with a custom python script, also adding our isolate and *Hamigera* *avellanea* sequences, with the latter as outgroup. The analysis was carried out through NGPhylogeny webservice [32] with a personalized workflow (https://ngphylogeny.fr/workflows/advanced/). The workflow consists in MAFFT [65] for the multiple alignment, BMGE [66] for alignment curation, FastTree [67] with GTR evolutionary model γ-distributed rate and 1000 as bootstrap value [68] for tree Interference and Newick Display [69] for tree rendering. In order to focus on the Citrina section, another tree was generated by the same method, i.e. considering the concatemer constituted of *BenA*, *CaM* and ITS sequences and adding the CBS strains of *P.* *sumatraense* available in NCBI that shared these genes. *RPB2* gene is unavailable for all these strains and therefore it was not considered. We selected only CBS strains because the CBS-KNAW culture collection is the largest one in the world with more 100,000 strains of fungi (including yeasts) and bacteria (https://wi.knaw.nl/page/Collection).

### Growth of *P. sumatraense* AQ67100 in liquid medium and determination of glycosyl hydrolase (GH) activities

*P.sumatraense* AQ67100 was grown in liquid medium at 20 °C on a rotary shaker (120 rpm) posed in a dark chamber. For liquid cultures, A-medium [0.6% (w/v) K_2_HPO_4_, 0.14% (w/v) (NH_4_)_2_SO_4_, 0.01% (w/v) MgSO_4_*7H_2_O, 0.2% (w/v) KH_2_PO_4_], and B-medium [0.6% (w/v) K_2_HPO_4_, 0.6% (w/v) (NH_4_)_2_SO_4_, 0.1% (w/v) MgSO_4_*7H_2_O, 0.6% (w/v) KH_2_PO_4_] were used. All the media were prepared fresh, pH-adjusted (6.5) and autoclaved. Three mycelium squares (5 × 5 mm) were cut out of a plate and inoculated in 50 ml of culture medium. After 2 days of growth, the cultures were supplemented with 0.2% (w/v) heat-treated *C.* *vulgaris* biomass and the growth prolonged up to 20 days. Supernatants from 20-day-old cultures were used as starting inoculum (1% v/v) for other cultures. The growth medium from algal-supplemented cultures was centrifuged (4000×*g*, 10 min) and the supernatant filtered using a sterile Filtropur 0.22 µm. Then, the filtered supernatants were dialyzed and concentrated (10×) using a Vivaspin 10,000 MWCO PES. Samples prepared according to this procedure were referred to as “filtrates”. Enzymatic activity was assayed by incubating the filtrate (10% v/v, 100 µl total volume) in 50 mM Na-acetate buffer pH 5 at 28 °C by using the following substrates: 1% (w/v) carboxymethyl-cellulose (CMC) to detect endo-β-1,4-glucanase activity, 1% (w/v) xyloglucan (XG) to detect xyloglucanase activity, 1% (w/v) arabinoxylan (AX) to determine arabinoxylanase activity, 0.3% (w/v) carboxymethyl-curdlan (CMCu) to detect endo-β-1,3-glucanase activity, 5 mM *p*-nitrophenyl-β-glucopyranoside (*p*NPG) to determine β-glucosidase activity, 5 mM *p*-nitrophenyl-β-galactopyranoside (*p*NPGal) to determine β-galactosidase activity and 5 mM *p*-nitrophenyl-β-laminaribioside (*p*NPL2) to determine exo-β-1,3-glucanase activity. All polysaccharides and *p*NPL2 were purchased from Megazyme (Bray, Ireland) whereas *p*NPG and *p*NPGal were purchased from Sigma-Aldrich (Saint Louis, USA). Enzyme activity was expressed as Enzyme Units (µmol of reducing sugar equivalents released per minute, or µmol of *p*-nitrophenol released per minute) per kg substrate or litre (L) culture. The amount of reducing ends released upon hydrolysis was determined according to [70] using different amounts of glucose as calibration curve. The amount of *p*-nitrophenol released upon hydrolysis was determined using different amounts of *p*-nitrophenol as calibration curve. Enzyme Units were expressed as mean of the values determined at two different time-points.

### Preparation of alcohol-insoluble solids (AIS) from *C. vulgaris*

Preparation of alcohol-insoluble solids (AIS) from *C.* *vulgaris* cells was performed according to [49] with some modifications. In brief, about 50 mg DW of *C.* *vulgaris* biomass was frozen in liquid nitrogen and homogenized with mortar and pestle. The resulting powder was washed three times in 70% (v/v) ethanol, vortexed, and pelleted by centrifugation (20,000×*g*, 10 min). The pellet was washed twice with a chloroform: methanol mixture [1: 1 (v/v)] and centrifuged (20,000×*g*, 10 min). The pellet was then washed three times with acetone up to significant discoloration and pelleted by centrifugation (20,000×*g*, 10 min). After evaporation of the solvent, the pellet was solubilized in B-medium and the resulting suspension used for downstream applications. Following this procedure, the AIS yield was about 40 ± 10% of the starting *C.* *vulgaris* biomass.

### Growth in different polysaccharide-supplemented media and enzymatic analysis

For growth experiments in different carbon source-supplemented media, B-medium was supplemented with 0.5% (w/v) AX, 0.5% (w/v) crystalline chitin (CHI), 0.5% (w/v) XG, 0.2% (w/v) heat-treated *C.* *vulgaris* biomass (C.v.) and 0.2% (w/v) *C.* *vulgaris* AIS (C.v. AIS). B-medium without any carbon source (NS) was used as negative control. Chitin from shrimp shells was purchased from Sigma-Aldrich (Saint Louis, USA). All polysaccharides were sterilized and supplied to B-medium (pH 6.5). Filtrates from culture media were prepared and assayed according to the procedure previously described. Alternatively, the filtrates were separated in a TGX™ Precast Protein Gel [4–15% (w/v) polyacrylamide] (Biorad, CA, USA) using Precision Plus Protein™ Dual Color Standards (Biorad, CA, USA) as molecular weight marker and then stained by silver nitrate. Proteolytic assay was performed by incubating 1 µg BSA with 20 µL of C.v.-filtrate for 16 h at 28 °C. BSA was purchased from Sigma-Aldrich (St. Louis, USA). The reaction was separated in 10% of Laemmli gel using Precision Plus Protein™ Dual Color Standards (Biorad, CA, USA) as molecular weight marker and then stained by silver nitrate.

### Protein identification by LC–MS/MS analysis

For protein identification analysis, 40 µL of 10× concentrated NS-, AX-, C.v.- and C.v. AIS-filtrates were incubated in Laemmli loading buffer at 100 °C and then loaded on a 10% acrylamide gel for separation by 1D-SDS-PAGE. Each lane was divided into ten different gel slices for in gel trypsin digestion [71]. Peptides were separated on a Pepmap C18 column (150 mm × 0.75 mm) at 300 nL min^−1^ with a 90 min multi step gradient of acetonitrile in 0.1% formic acid, using an Ultimate 3000 nano-chromatography pump (Thermo-Fisher Scientific) coupled to an LTQ Orbitrap Discovery mass spectrometer (Thermo-Fisher, Bremen, Germany) operated in a data dependent mode. MS was acquired at 30.000 FWHM resolution in the FTMS (using a target value of 5 × 10^5^ ions) and MS/MS was carried out in the linear ion trap. Five MS/MS scans were obtained per MS cycle. The raw data from the mass spectrometric analysis were processed using the MaxQuant software v. 1.6.17 [72] supported by Andromeda as the database search engine for peptide identifications, using a protein database constructed ad hoc from the annotated genome of *P.* *sumatraense* AQ67100. The main peptide identification parameters were the following: trypsin cleavage specificity, variable methionine oxidation and N-term acetylation, cysteine carbamidomethylation as fixed modification and mass tolerance for parent and fragment ions of ± 20 ppm and ± 0.5 Da, respectively.

Protein identification was performed in the MaxQuant Identify module using the following parameters: protein and peptide false discovery rate (FDR) < 0.01, posterior error probability based on Mascot score, minimum peptide length of 7.

### Enzymatic treatment of *C. vulgaris* cells and determination of metabolites

For the enzymatic treatment, 0.33 mg of *C.* *vulgaris* biomass was washed, heat-treated (70 °C, 50 min) and incubated with 0.3 mL of different enzymatic mixtures. The enzymatic mixtures consisted of (i) a recombinant cellulase from *Aspergillus* *niger* (0.24 U mL^−1^), (ii) a blend composed of lysozyme from chicken hen egg white (100,000 U mL^−1^), chitinase from *Streptomyces* *griseus* (0.15 U mL^−1^) and sulfatase H1 from *Helix* *pomatia* (10 U mL^−1^) according to [17] and (iii) a filtrate (10X) of *P.* *sumatraense* AQ67100 as obtained from a 10-day-old *C.* *vulgaris-*supplemented culture (0.13 ± 0.03 U mL^−1^ of β-glucosidase activity). Lysozyme, chitinase and sulfatase were purchased from Sigma-Aldrich (St. Louis, USA) whereas the cellulase was purchased from Megazyme (Bray, Ireland). All the enzyme mixtures were prepared fresh, filter-sterilized and dialyzed using B-medium as buffer exchange. The reactions were incubated at 28 °C for 16 h. Following the incubation, the reaction mixtures were centrifuged (14,000×*g*, 10 min) and the amount of sugars, chlorophylls and lipids spectrophotometrically determined in the supernatants. Determination of reducing and total sugars was performed in accordance with [70] and [73], respectively, using different amounts of glucose to build a calibration curve. Determination of chlorophylls was performed in accordance with [74]. Determination of lipids was performed by the sulfo-phospho-vanillin (SPV) assay according to Mishra et al. (75).

Extraction of metabolites from enzymatically treated *C.* *vulgaris* cells was performed by using 1 mL of 60% (v/v) ethanol or 1.5 mL of [DMSO: acetone: H_2_O] (10: 80: 10, v: v: v). The chlorophyll yield obtained by using [DMSO: acetone: H_2_O] was reported as reference. Determination of lipids in both ethanolic extracts and raw *C.* *vulgaris* biomass was performed by SPV assay. The lipid yield obtained from raw *C.* *vulgaris* biomass was reported as reference. All the spectrophotometric determinations were performed by using an Infinite^®^ M Nano200 spectrophotometer (Tecan AG, Männedorf, Switzerland).

## Supplementary Information

**Additionalfile1:Figure S1**. Design of the algal trap. **Figure S2**. Morphological characteristics of the unknown fungal isolate. **Figure S3**. Extraction of genomic DNA (gDNA) from the unknown fungal isolate. **Figure S4**. Growth of *P.sumatraense* AQ67100 in different algal-supplemented media. **Figure S5**. Time-course analysis of GH activities from *P.* *sumatraense* AQ67100 cultures as grown in algal-supplemented media. **Figure S6**. Analysis of GH activities from *P.* *sumatraense* AQ67100 cultures upon growth in different cell wall polysaccharide-supplemented media. **Figure S7**. Evaluation of protease activity in C.v.-filtrate. **Figure S8**. Protein bands putatively ascribable to the main algal-degrading enzymes. **Figure S9**. Growth of *P.* *sumatraense* AQ67100 in a chitin-supplemented medium.

**Additionalfile2:Data S1–S3**. Sequences of predicted genes (Data S1), CDSs (Data S2) and proteins (Data S3) as obtained from the annotated genome of the unknown fungal isolate (FASTA format). **Data S4**. GTF (Gene Transfer format) obtained by BRAKER annotation with gene structure and coordinates. **Data S5**. Functional annotations of predicted proteins (TXT format). **Data S6**. Phylogenetic markers used for the classification of the unknown fungal isolate.

## Data Availability

All relevant data are included in the article and/or its Additional files. The sequence reads of *P.* *sumatraense* AQ67100 genome have been deposited at the NCBI Sequence Read Archive (SRA) under the BioProject ID PRJNA716633 whereas the corresponding assembly data set has been deposited at GenBank under the accession number JAGIKU000000000.

## Abbreviations

AIS: Alcohol-insoluble solids
AX: Arabinoxylan
CHI: Chitin
CMC: Carboxymethyl-cellulose
CMCu: Carboxymethyl-curdlan
C.v.: *Chlorella* *vulgaris*
CWDE: Cell wall-degrading enzyme
GH: Glycosyl hydrolase
Phi: Phosphite
Pi: Phosphate
*p*NPG: *p*-Nitrophenyl-β-glucopyranoside
*p*NPGal: *p*-Nitrophenyl-β-galactopyranoside
*p*NPL2: *p*-Nitrophenyl-β-laminaribioside
XG: Xyloglucan
