# Supplementary material for: A novel Penicilliumsumatraense isolate reveals an arsenal of degrading enzymes exploitable in algal bio-refinery processes
Source: Biotechnol Biofuels. 2021 Sep 13;14:180. doi: 10.1186/s13068-021-02030-9 (PMC8438893; doi:10.1186/s13068-021-02030-9)
Supplement: Supplementary file 1 — Additionalfile1:Figure S1. Design of the algal trap. Figure S2. Morphological characteristics of the unknown fungal isolate. Figure S3. Extraction of genomic DNA (gDNA) from the unknown fungal isolate. Figure S4. Growth of P.sumatraense AQ67100 in different algal-supplemented media. Figure S5. Time-course analysis of GH activities from P. sumatraense AQ67100 cultures as grown in algal-supplemented media. Figure S6. Analysis of GH activities from P. sumatraense AQ67100 cultures upon growth in different cell wall polysaccharide-supplemented media. Figure S7. Evaluation of protease activity in C.v.-filtrate. Figure S8. Protein bands putatively ascribable to the main algal-degrading enzymes. Figure S9. Growth of P. sumatraense AQ67100 in a chitin-supplemented medium. [file 13068_2021_2030_MOESM1_ESM.pdf]

a) b)

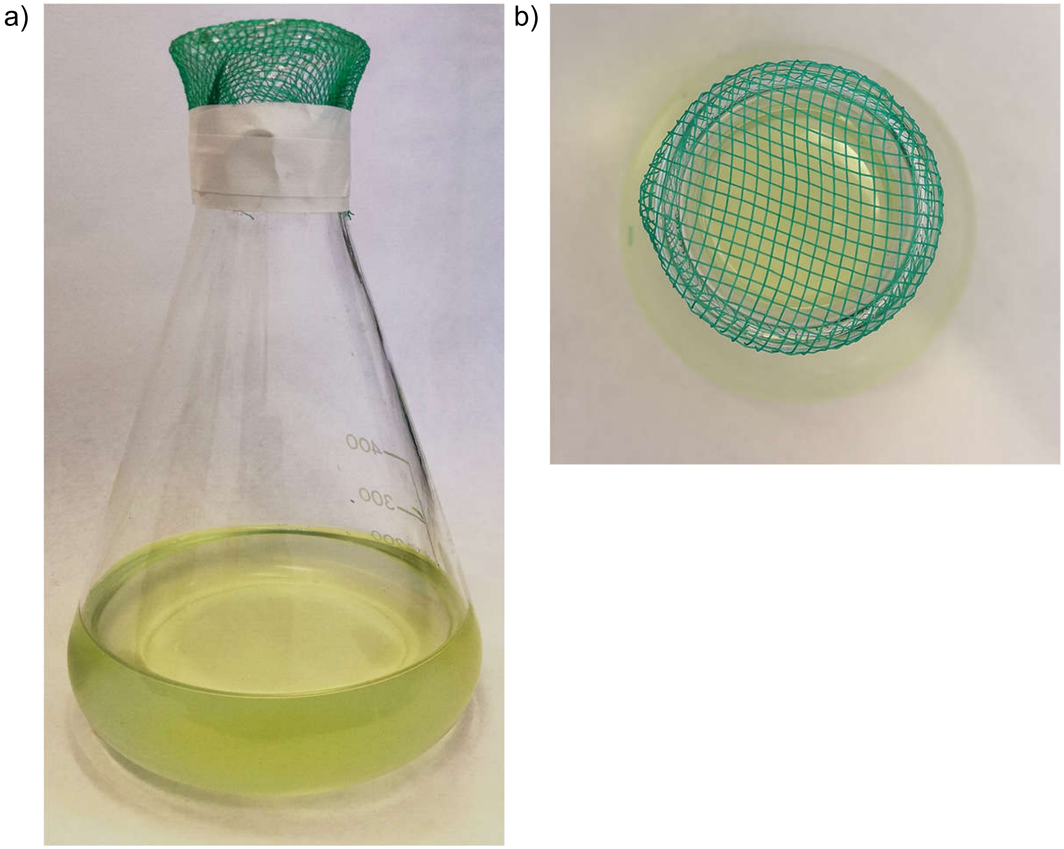

Figure S1. Design of the algal trap. a) Image of a freshly prepared algal trap. b) Top view of the algal trap.

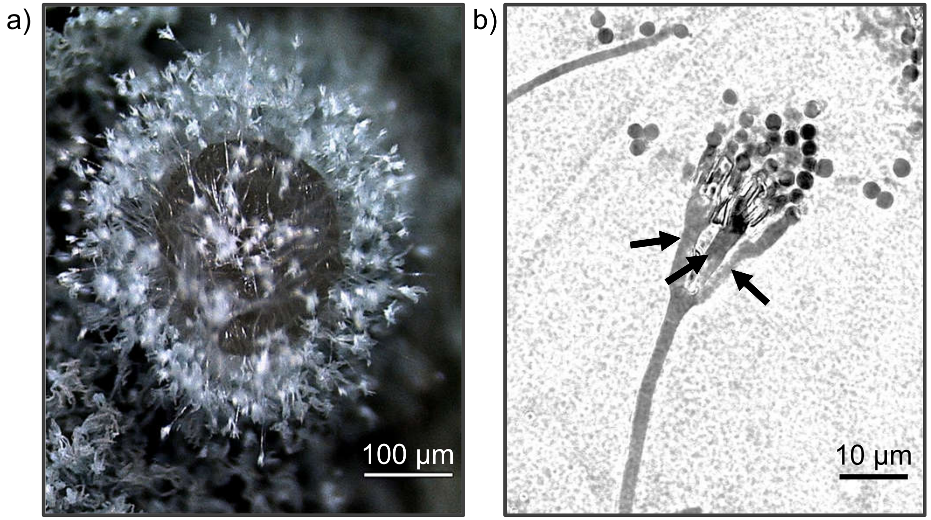

Figure S2. Morphological characteristics of the unknown fungal isolate. a) Conidial head of the fungus as grown on solid MEP medium by stereo microscope analysis (8X magnification). b) Conidiophore and conidia of the fungus as grown on solid MEP medium by optical microscope analysis (100X magnification). Black arrows point to three different metulae. Scale bars are also indicated.

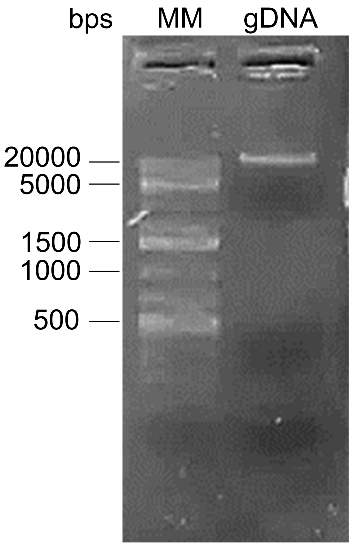

Figure S3. Extraction of genomic DNA (gDNA) from the unknown fungal isolate. a) Agarose gel electrophoresis of fungal gDNA. The molecular weight marker (MM: GeneRuler 1 kb Plus DNA Ladder) is also shown; only some molecular weights are reported.

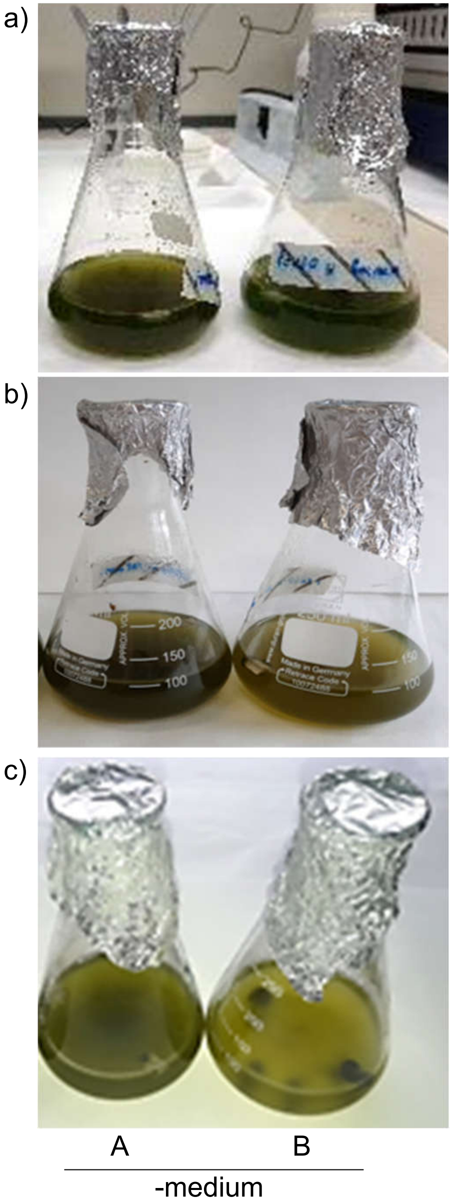

Figure S4. Growth of *P. sumatraense* AQ67100 in different algal-supplemented media. Fungal cultures as grown in A- and B-medium supplemented with 0.2 % (w/v) heat-treated *C. vulgaris* biomass at a) 2, b) 4 and c) 7 days from algal administration.

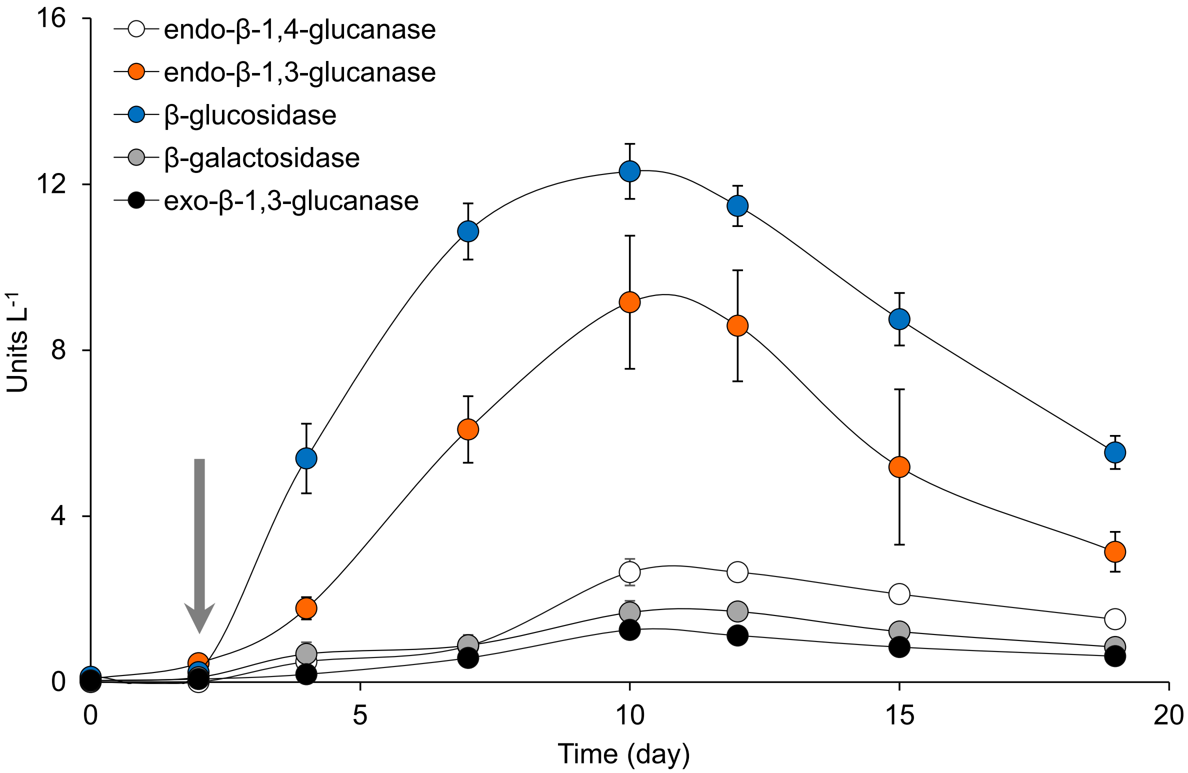

Figure S5. Time-course analysis of GH activities from *P. sumatraense* AQ67100 cultures as grown in algal-supplemented media. GH activities were evaluated in the filtrates from algal-supplemented cultures upon different days of growth. B-medium was supplemented with 0.2% (w/v) heat-treated *C. vulgaris* biomass upon two days of growth (grey arrow). Units are expressed as µmol reducing ends (for endo-β-1,4-glucanase and endo-β-1,3-glucanase activities) and µmol *p*nitrophenol (for β-glucosidase, β-galactosidase and exo-β-1,3-glucanase activities) released per minute. Arabinoxylanolytic and xyloglucanolytic activities were not significantly detected. Experiments were repeated twice with consistent results.

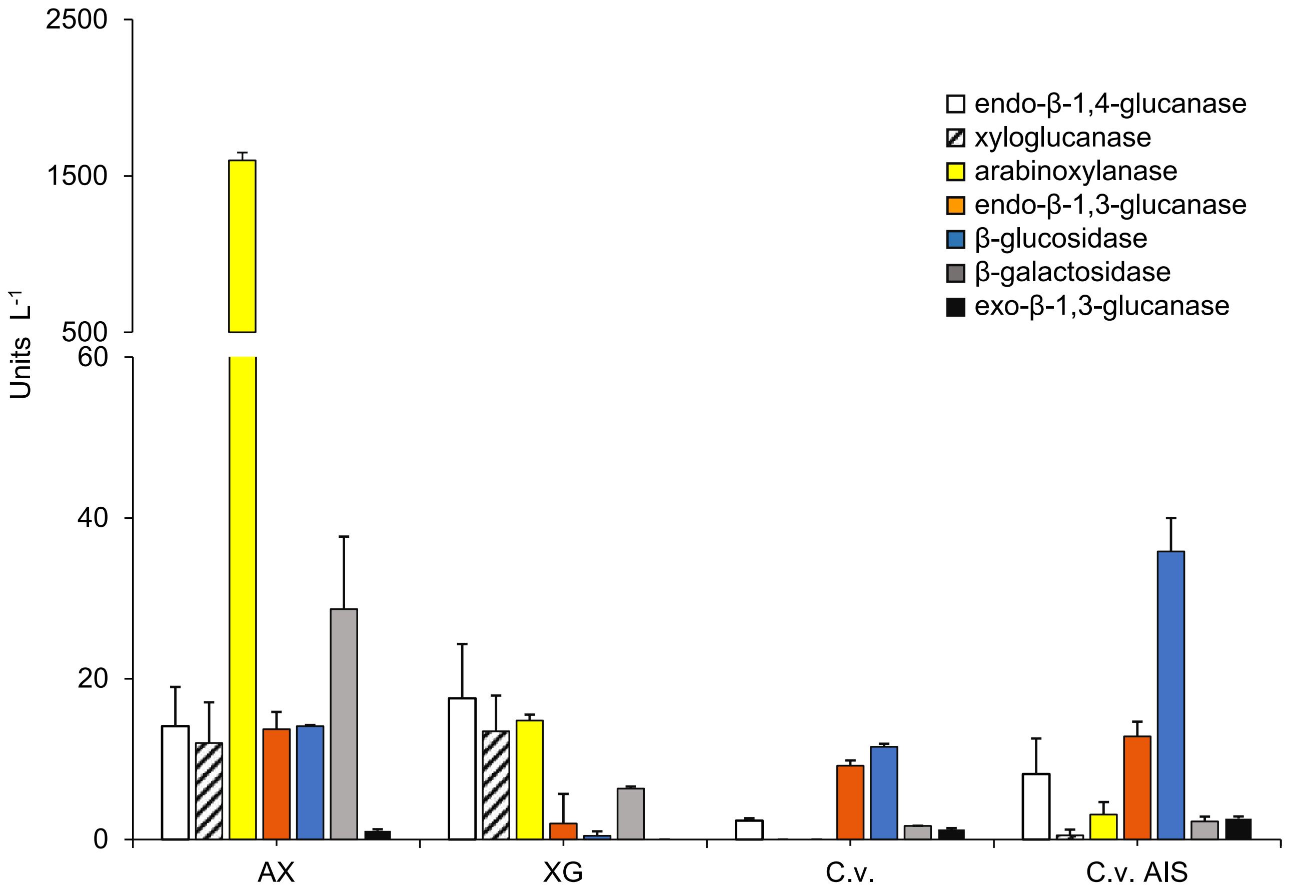

Figure S6. Analysis of GH activities from *P. sumatraense* AQ67100 cultures upon growth in different cell wall polysaccharide-supplemented media. a) Analysis of GH activities in filtrates from different cell wall polysaccharide-supplemented cultures upon 10 days of growth. B-medium was supplemented with 0.5% (w/v) arabinoxylan (AX), 0.5% (w/v) xyloglucan (XG), 0.2% (w/v) heat-treated *C. vulgaris* biomass (C.v.) and 0.2% (w/v) *C.vulgaris* AIS (C.v. AIS). Units are expressed as μmol reducing ends (for endo-β-1,4-glucanase, xyloglucanase, arabinoxylanase and endo-β-1,3-glucanase activities) and μmol *p*nitrophenol (for β-glucosidase, β-galactosidase and exo-β-1,3-glucanase activities) released per minute. Experiments were repeated in triplicate with consistent results.

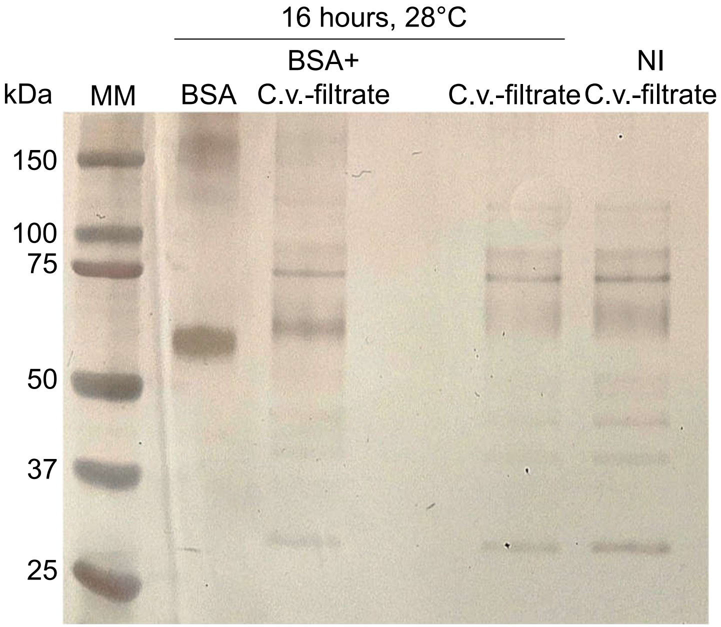

Figure S7. Evaluation of protease activity in C.v.-filtrate. BSA was incubated with C.v.-filtrate for 16 hours at 28°C (BSA + C.v.-filtrate) and the reaction analyzed by SDS-PAGE. An equivalent amount of 200 ng of BSA and 4 µL of C.v.-filtrate was analyzed [BSA, bovine serum albumin; NI, not incubated]. The molecular weight marker (MM) is also reported.

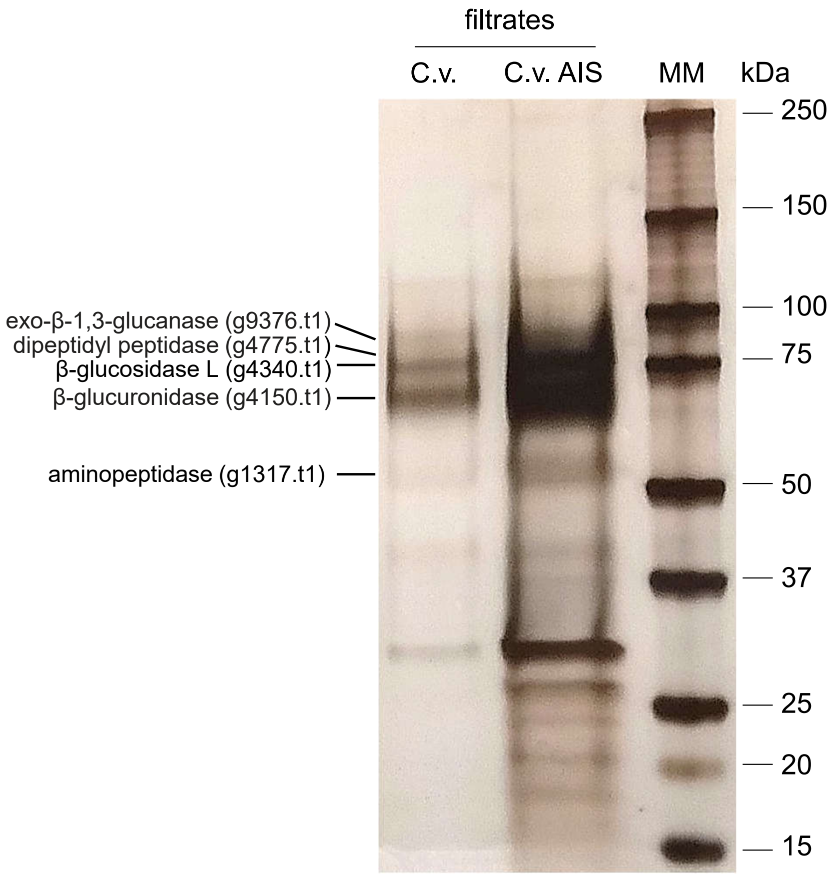

Figure S8. Protein bands putatively ascribable to the main algal-degrading enzymes. Detail of the SDS-PAGE analysis shown in Figure 5b including (*left side*) the approximate band position (height) of the most prominent enzymes from C.v.- and C.v. AIS-filtrates as determined by LC-MS/MS analysis. The molecular weight marker (MM) is also reported. Further details on the identified proteins are reported in Table 4 and Figure 6.

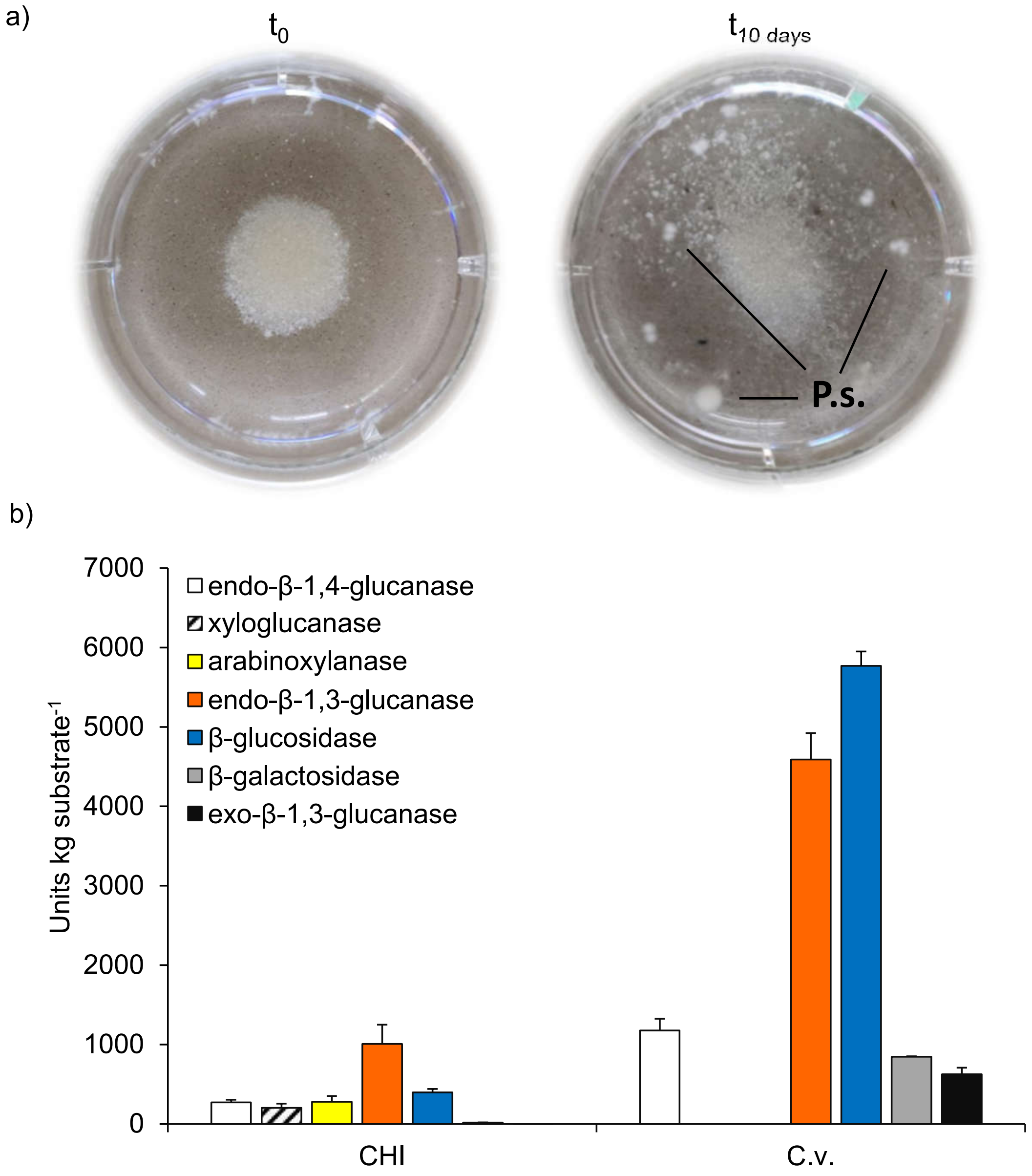

Figure S9. Growth of *P. sumatraense* AQ67100 in a chitin-supplemented medium. a) Fungal culture as grown in B-medium supplemented with 0.5 % (w/v) crystalline chitin (CHI) at 0 and 10 days of incubation [P.s., *P. sumatraense* AQ67100]. b) GH activities in filtrate from the 10-days old culture shown in a). GH activities in filtrate from the culture supplemented with 0.2% (w/v) heat-treated *C. vulgaris* biomass (C.v.) are reported as reference. Units are expressed as μmol reducing ends (for endo-β-1,4-glucanase, xyloglucanase, arabinoxylanase and endo-β-1,3-glucanase activities) and μmol *p*nitrophenol (for β-glucosidase, β-galactosidase and exo-β-1,3-glucanase activities) released per minute. Experiments were repeated twice with consistent results.
