# Supplementary material for: A novel Penicilliumsumatraense isolate reveals an arsenal of degrading enzymes exploitable in algal bio-refinery processes
Source: Biotechnol Biofuels. 2021 Sep 13;14:180. doi: 10.1186/s13068-021-02030-9 (PMC8438893; doi:10.1186/s13068-021-02030-9)
Supplement: Supplementary file 2 — Additionalfile2:Data S1–S3. Sequences of predicted genes (Data S1), CDSs (Data S2) and proteins (Data S3) as obtained from the annotated genome of the unknown fungal isolate (FASTA format). Data S4. GTF (Gene Transfer format) obtained by BRAKER annotation with gene structure and coordinates. Data S5. Functional annotations of predicted proteins (TXT format). Data S6. Phylogenetic markers used for the classification of the unknown fungal isolate. [file 13068_2021_2030_MOESM2_ESM.zip › Data_S6.docx]

**Data S6. Phylogenetic markers used for the classification of the unknown fungal isolate*.*** Sequences of the four genetic markers used for the phylogenetic analysis shown in Figure 2 as obtained from the annotated genome of the unknown fungal isolate, i.e., the genes encoding the RNA polymerase II second largest subunit (*RPB2*, gene ID: g10996), β-tubulin (*BenA*, gene ID: g5792) and calmodulin (*CaM*, gene ID: g2696) together with the Internal Transcribed Spacer (ITS) region. Introns are reported in lowercase.

*RPB2*

ATGGCCGACTATGACGAAGCTTATGAGGATGAGTTTTACGACGACGCGGATGAGGGTATCACCTCTGAGGACTGCTGGTCCGTGATCTCTTCCTTCTTCGATACCAAGGGTCTTGTGTCACAGCAACTCGACTCTTTCGATGAATTCATCTCTTCGACGATGCAGGAACTTGTGGAGGAACAAGGACAAGTGGTTCTCGACCAGACACTTCCGCCCGCCGAAGACGAAATCGACCCGGTCGTCGTTCGCCGTTATGAGCTCAAATTCGGAACCATCATGCTTTCGCGACCATCCGTGACGGAAGGCGATGGCGCTACGACAATCATGTTGCCGCAAGAAGCTCGCCTCCGAAACTTGACTTACGCCAGTCCGTTGTACCTGAACGTCTCAAAGAAGATTATGGAGGGCCGAGAGCGCATGGTGGGTGATGGCGATGAGGATGAGGGCGAGGCGGATGAAGACCGCAAGAACCGTGGAACATACCTGCAATGGGAGACAAAGGAACTACCAGAGCAGCAAAGGAAAGAGGATACAGTTTTCATTGGAAAGATGCCCATCATGCTGAAATCCAAGTACTGTATCTTGAAGGACTTGAACGAGCAGGCTCTTTACGCTTGGAACGAGTGTCCCTACGATTCCGGTGGCTACTTTATCATCAACGGAAGTGAAAAGGTTCTGATCGCACAGGAGCGCAGTGCTGGTAACATTGTGCAAGTCTTCAAGAAAGCGCCCCCGAGTCCCACACCCTACGTTGCCGAAATCCGAAGTGCCGTCGAGAAGGGATCCCGACTCCTGTCGCAACTTGCCCTCAAGCTATTCGCCAAGGGTGACAGTGCCAAGGGCGGATTCGGTCCGACTATTCGCTCCACCCTTCCCTACGTCAAGGCCGATATTCCCATTGTCGTTGTTTTCCGAGCCCTCGGCGTTGTCTCCGATGAAGATATCCTTAACCACATTTGCTACGACCGCAATGATACCCCTATGCTAGAGATGCTCAAGCCGTGTATTGAGGAAGGATTCGTCATTCAAGACCGAGAAGTCGCTCTGGATTTCATCGCCAAGCGTGGCTCTTCCCAGTCCAACCTCAACCACGAGCGTCGTGTGCGCTACGCCCGGGAGATTATGCAGAAGGAACTGCTGCCTCACATCTCGCAGAGCGAGGGCAGTGAAACCCGTAAGGCTTTCTTCTTGGGTTACATGGTTCACCGTCTTCTCCAGTGTGCCCTTGGCCGCCGTGACGTCGACGACCGTGATCACTTCGGAAAGAAGCGTCTTGATCTCGGTGGTCCTCTTCTTGCCAACCTTTTCCGTGTCCTTTTCACTCGTGTCACTCGCGATCTGCAGCGTTATGTCCAACGGTGTGTTGAGACCAACCGTGAGATCTACTTGAACATTGGTCTCAAGGCCGCTACCTTGACCGGTGGTTTGAAGTATGCCCTTGCTACAGGTAACTGGGGTGAACAGAAGAAGGCAGCCAGCGCTAAGGCCGGTGTGTCCCAAGTGCTGAGTCGCTACACATTCGCTTCTTCGCTGTCGCATTTGCGCCGTACCAACACGCCTATTGGTCGTGATGGTAAGATTGCCAAACCGCGTCAACTTCATAATACCCACTGGGGTCTGGTGTGTCCCGCAGAAACACCCGAAGGACAGGCTTGTGGTCTTGTCAAGAACTTGGCACTTATGTGCTATATCACTGTTGGTACTCCTAGTGAGCCCATCATCGATTTCATGATTCAGCGAAATATGGAAGTCTTGGAAGAGTTTGAACCTCAAGTCACGCCGAATGCCACAAAGGTCTTCGTTAATGGTGTCTGGGTTGGTATCCACCGTGATCCTTCTCACCTGGTTAACACTATGCAGTCCCTGCGTCGACGCAACATGATTTCTCACGAAGTCAGTTTGATTCGGGATATTCGTGAGCGAGAGTTCAAGATTTTCACCGATACCGGCCGTGTCTGCCGCCCCTTGTTCGTTGTCGACAATGATCCCAAGAGCGAAAATGCGGGATCTTTGATTCTGAATAAGGAGCACATTCACAAGCTGGAACAGGACAAGGACCTGCCTCTAGATATGGATGTTGAAGAGCGACGAGAGCGCTACTTCGGATGGGATGGTCTGGTTCGATCAGGAGCTGTTGAGTATGTCGATGCGGAAGAAGAAGAGACTATAATGATTGTGATGACGCCTGAAGATTTGGAGATTTCCAAGCAGCTCCAGGCCGGCTACGCATTGCCCGAGGAAGAGTCCAGTGACCCGAACAAGCGAGTCCGCTCGATTCTCAGCCAGCGGGCGCACACCTGGACACACTGTGAGATTCATCCCAGTATGATTCTCGGTGTTTGTGCCAGTATTATTCCTTTCCCGGATCACAACCAGTCTCCTCGTAACACTTACCAGTCTGCCATGGGTAAGCAGGCCATGGGTGTTTTCCTGACAAACTTTGCCCAGCGCATGGAGACCATGGCCAATATTCTCTACTACCCCCAGAAGCCTCTGGCCACAACTCGATCAATGGAGTTCTTGCGCTTCCGCGAGCTTCCTGCTGGACAGAACGCCATTGTCGCCATTGCCACTTACTCCGGTTACAACCAAGAAGATTCCGTTATTATGAACCAGAGCAGTATCGATCGTGGACTGTTCCGCAGTTTGTTCTACCGTACATACACCGATTCCGAGAAGATGGTTGGTTTGACAGTTGTCGAGCGATTCGAGAAGCCCATGCGCTCCGACACAATTGGTATGCGCAAGGGTACCTACGACAAGTTGGACGAGGATGGTATTATCGCCCCTGGTGTTCGTGTTTCCGGAGAAGATATCATCATCGGAAAGACCGCACCTTTGGCAGCCGATGCAGAGGAGCTTGGTCAACGTACCAAGGCACACACCAAGATCGATGTGTCGACGCCACTGCGAAGTACCGAGAACGGTATTGTGGATCAGGTCTTGATCTCTACTGGCAATGACGATCTCAAATTCGTCAAGGTCCGTATGCGTACCACAAAGGTTCCCCAGATTGGTGACAAGTTCGCGTCTCGTCACGGTCAAAAGGGTACCATTGGTATCACCTACCGACAGGAGGATATGCCTTTCACTCGCGAGGGTGTTGTCCCCGATCTGATTATCAACCCACACGCCATTCCCTCTCGTATGACTATTGCTCACTTGATCGAGTGTCAATTGAGTAAGGTCTCAGCTCTCCGTGGTTTCGAAGGTGATGCCACTCCATTCACCGATGTCACTGTCGACTCCATCTCGCGTCTACTGCGCGAGCACGGTTACCAATCTCGTGGTTTCGAAGTTATGTTCAACGGCCATACCGGTCGCAAGCTTGTTGCACAGGTCTTCCTGGGTCCAACCTACTACCAGCGTCTTCGTCACATGGTAGACGACAAGATTCACGCTCGTGCTCGTGGCCCTACCCAAATTCTCACCCGTCAACCCGTCGAAGGTCGTGCACGTGATGGTGGTCTGCGTTTCGGAGAGATGGAACGCGATTGCATGATTGCCCATGGTGCCAGTGCCTTCTTGAAGGAGCGTCTCTTCGATGTTTCCGATCCTTTCCGCGTTCACATTTGCGACGACTGCGGTCTGATGACCCCTATTGCgtaagttctacatccctatctcagctgcatctctgcatctcttcttggtataccgacatcttactaacacatcaacagAAAACTGAAGAAGGGTCTCTTCGAGTGCCGTCTCTGCAATAACAAACACCGCATCTCACAGGTCCACATCCCCTACGCCGCCAAGCTTCTGTTCCAAGAGCTGGCCTCGATGAACATTGCCGCTCGGATGTTTACCGACCGGTCGGGTGTATCGGTGCGATAA

*BenA*

ATGCGTGAGATCgtacgtcctcttgtcccaagttcaacgcgtctttgttgacctacccctgaacggacccccacttgttcatcctgctaacctgagctttttttccccaacgtatagGTTCACCTTCAGACCGGCCAGTGTgtaagtgcacattcgaagaatccaatgccctcatgatcgggcaaaaagacacaactatatgagtatgatggttcgaatgaatgctaaatgatatgtggggacatacagGGTAACCAAATTGGTGCCGCTTTCTGgtacgtgctgcaatcccaaaacaatcaattgttgaatgcatgaagcaataaactaatcaattcaacagGCAAACCATTGCTGGCGAGCACGGCCTTGATGGCGATGGACAgtgagtgatttcgaccaggttttgattttcgagaatggcggtctgatatttttgggcagCTACAACGGTACCTCTGACCTCCAGCTGGAGCGCATGAACGTTTACTTCACCCATgtaagcgacaacaatcccatcaatacaattagtcttgattctaacggcttgtttttctgtttacaatagGCTTCCGGTGACAAGTATGTTCCCCGTGCCGTTCTGGTTGATCTGGAGCCCGGTACCATGGACGCTGTCCGTGCCGGTCCCTTCGGCAAGCTCTTCCGCCCCGACAACTTCGTCTTCGGTCAGTCTGGTGCTGGTAACAACTGGGCCAAGGGTCACTACACTGAGGGTGCCGAGCTCGTTGACCAGGTCGTCGATGTCGTCCGCCGTGAGGCCGAGGCTTGCGACTGCCTCCAGGGTTTCCAGATCACCCACTCCCTGGGTGGTGGTACCGGTGCCGGTATGGGTACACTCCTGATCTCCAAGATCCGTGAGGAGTTCCCCGACCGTATGATGGCCACCTTCTCCGTTGTTCCCTCCCCCAAGGTCTCGGATACCGTTGTCGAGCCTTACAACGCTACCCTGTCCGTTCACCAGCTGGTTGAGCACTCCGACGAGACTTTCTGTATCGATAACGAGgtatggatagtggcctatgaatgccctacgaaaacagccaattgactaatttcatgatagGCTCTGTACGACATCTGCATGCGCACCCTTAAGCTGTCTCAGCCCTCCTACGGTGACCTGAACCACCTGGTCTCTGCCGTCATGTCCGGTGTCACCACCTCGCTCCGTTTCCCCGGTCAGCTCAACTCCGATCTCCGCAAGCTGGCTGTCAACATGGTTCCTTTCCCTCGTCTCCACTTCTTCATGGTTGGATTCGCTCCCCTGACCAGCCGTAACGCCAATGCCTACCGCCAGGTCAGCGTTCCCGAGCTGACCCAGCAGATGTTCGACCCCAAGAACATGATGGCTGCTTCTGACTTCCGTAACGGCCGTTACCTCACCTGCTCCGCTCTGTTCCGCGGTAAGGTCTCCATGAAGGAGGTCGAGGACCAGATGCGCAGCATCCAGACCAAGAACCAGAGCTACTTCGTCGAGTGGATTCCCAACAATGTCCAGACCGCCCTGTGCTCCGTTCCTCCCCGCGGCCTGCGCATGTCCTCCACCTTCGTCGGTAACAGCACCTCTATCCAGGAGCTGTTCAAGCGTATCGGTGACCAGTTCACTGCCATGTTCCGCCGCAAGGCTTTCTTGCACTGGTACACTGGTGAGGGTATGGACGAGATGGAGTTCACTGAGGCTGAGAGCAACATGAACGACCTGGTCTCTGAATACCAGCAATACCAGGATGCCTCCATCTCCGAGGGTGAGGAGGAGTACCTCGCTGAGGAGGCTGCTCTCGAGGATGAGGTCTAA

*CaM*

ATGgtatgtctcttacttgacacctctttggcctcactactaccccctgttatgtatctcggggtgcaggacctcccatcccgagacacttcgcactgcggcataccccattgttgtcccgaatgttgttgactaactgcgccatcactgctaaatagGCCGATTCTCTGACTGAAGAGCAAGTTTCCGAGTACAAGGAGGCGTTCTCCCTCTTTgtgagtaattcgatccaggaattgaaattgtgaacgtgtgatcgattttaggctgacggggggttatcttgtgatcgacagGACAAGGATGGTGATGgtgagttctgtcgtttgtgaacgacccgtgtcctttctacgggcggtgtttctcttgcgattcgtttccatcaaaattcactcgaaatatactgattgatcgatgataaatagGACAAATCACCACCAAGGAGCTCGGCACTGTCATGCGCTCCCTCGGCCAGAACCCCTCCGAGTCCGAGCTACAGGACATGATCAATGAGGTCGATGCCGATAACAATGGCACCATTGATTTCCCTGgtacgaatcccagagtccatattcctatgactccctctcttcacctacattatgccccatcgtcgcatctcgatacgaaagaaataaatattgacatgcgcccacagAGTTCCTGACCATGATGGCTCGTAAGATGAAGGATACCGACTCCGAGGAGGAGATCCGCGAGGCTTTCAAGGTGTTTGATCGCGATAACAACGGATTCATCTCTGCCGCTGAGCTGCGCCACGTCATGACCTCCATCGGCGAGAAGCTGACCGACGATGAGGTCGATGAGATGATCCGTGAGGCCGATCAGGACGGCGACGGCCGTATCGACTgtacatatcccccgctcccgcatcctggaatttctcgagaggggacatgctaacaattctcttttttagACAACGAGTTCGTCCAGCTCATGATGCAAAAATAA

ITS

CTGAGTGAGGGCCCCTCGGGGTCCAACCTCCCACCCGTGTTTAACGAACCTTTGTTGCTTCGGCGGGCCCGCCTCACGGCCGCCGGGGGGCTCCTGCCCCCGGGCCCGCGCCCGCCGAAGCCCCCCCTTGAACGCTGTCTGAAGTTTGCAGTCTGAGAAACTAGCTAAATTAGTTAAAACTTTCAACAACGGATCTCTTGGTTCCGGCATCGATGAAGAACGCAGCGAAATGCGATAACTAATGTGAATTGCAGAATTCAGTGAATCATCGAGTCTTTGAACGCACATTGCGCCCTCTGGTATTCCGGAGGGCATGCCTGTCCGAGCGTCATTGCTGCCCTCAAGCACGGCTTGTGTGTTGGGCCCCCGTCCCCCCCTCTGCCGGGGGGACGGGCCCGAAAGGCAGCGGCGGCACCGCGTCCGGTCCTCGAGCGTATGGGGCTTCGTCACCCGCTCTTGTAGGCCCGGCCGGCGCCAGCCGACCCCAACCCTAAATTTTTTTCAG
